# Contributions of a small collection of the terrestrial microsnails genus *Diplommatina* s.l. (Caenogastropoda, Cyclophoroidea, Diplommatinidae) from Myanmar, with a catalogue of recorded species and description of two new species

**DOI:** 10.3897/zookeys.1285.186884

**Published:** 2026-07-17

**Authors:** Piyoros Tongkerd, Ngwe Lwin, Jonathan D. Ablett, Arthit Pholyotha, Supunya Annate, Chirasak Sutcharit

**Affiliations:** 1 Animal Systematics Research Unit, Department of Biology, Faculty of Science, Chulalongkorn University, Bangkok 10330, Thailand Animal Systematics Research Unit, Department of Biology, Faculty of Science, Chulalongkorn University Bangkok Thailand; 2 Fauna and Flora International, No. 35, 3rd Floor, Shan Gone Condo, Myay Ni Gone Market Street, Sanchaung Township, Yangon, Myanmar Mollusca Section, Invertebrates Division, Department of Life Sciences, The Natural History Museums London United Kingdom; 3 Mollusca Section, Invertebrates Division, Department of Life Sciences, The Natural History Museums, London SW7 5BD, UK Fauna and Flora International Yangon Myanmar

**Keywords:** Conservation, limestones, micro-CT, Southeast Asia, taxonomy

## Abstract

All currently known microsnails of the genus *Diplommatina* s.l. from Myanmar are here listed and revised based on recently collected specimens. The specimens examined were obtained through the ‘Conserving Myanmar’s Karst Biodiversity’ project, hosted by the Fauna & Flora International in collaboration with the Forest Department of Myanmar. In total, 31 nominal species are documented from the country. Among these, three species originally described from this region, namely *D. carneola*, *D. scalaroidea*, and *D. richthofeni*, were rediscovered and re-described. We also report on three new country records, *D. akron*, *D. nimanandhi*, and *Pagodapalaina suratensis* [formerly *Diplommatina*], previously known only from Thailand. In addition, two new species are described based on distinctive morphological characters: *D. prolixa* Tongkerd, **sp. nov**. from Kayin State and *D. somsakpanhai* Tongkerd, **sp. nov**. from the Tanintharyi Region. For twenty-nine taxa, we examined and illustrated name-bearing types and/or authenticated specimens whenever available, as these are critical for systematic revision. Overall, this study provides updated knowledge of the *Diplommatina* s.l. in Myanmar and offers a valuable foundation for future taxonomic and systematic research in Indochina. To stabilise nomenclature, *D. stanisici* Tongkerd, **nom. nov**., is herein proposed as replacement name for the Australian species *Diplommatina angulata* Stanisic, 2010, which is a junior primary homonym of *D. angulata* Theobald & Stoliczka, 1872 from Myanmar.

## Introduction

The terrestrial microsnail genus *Diplommatina* Benson, 1849, renowned for its distinctive and intricate shell morphology, represents a highly speciose genus with a broad distribution extending across Southeast Asia as well as south China, Japan, the Indo-Pacific islands, and northern Australia (Kobelt 1902; Gude 1921; Vermeulen 1993; Panha and Burch 2005; Egorov 2013; Greke et al. 2023). Members of the genus are typically characterised by their minute shells (usually < 5 mm in length), which may be dextral or sinistral, more or less conical in shape, and commonly possessing internal lamellae (Egorov 2013; Budha et al. 2017; Nurinsiyah and Hausdorf 2017; Vermeulen and Liew 2022). In recent years, this microsnail genus has received increasing attention, with numerous classical taxonomic studies describing new taxa (e.g., Do et al. 2015; Foon et al. 2017; Vermeulen et al. 2019; Dumrongrojwattana et al. 2020). In addition, several phylogenetic studies have been published that contribute to a better understanding of the evolutionary relationships among the *Diplommatina* s.l. (Rundell 2008; Webster et al. 2012; Boonmachai et al. 2023; Köhler 2023; Páll-Gergely et al. 2024). However, these recent investigations have focused primarily on the Indochinese countries and have mostly excluded Myanmar.

However, the microsnails of Myanmar should not be ignored, as it is the largest country in mainland Southeast Asia, and lies within the Indo-Burma biodiversity hotspot and contains extensive limestone karst landscapes (Myers et al. 2000; Mittermeier et al. 2004; Asian Development Bank 2012; Tordoff et al. 2012). During the past two decades, research on various groups of land snails in Myanmar has gradually advanced, with systematic revisions published for some caenogastropod families (e.g., Páll-Gergely et al. 2014, 2015, 2021; Páll-Gergely and Grego 2019; Tongkerd et al. 2023, 2024; Jirapatrasilp et al. 2025) and various heterobranch families (e.g., Páll-Gergely 2018; Páll-Gergely et al. 2020, 2022; Pholyotha et al. 2020, 2022; Sutcharit et al. 2020a, 2020b; Sutcharit and Panha 2021; Man et al. 2022, 2023, 2024; Tongkerd et al. 2023), many of which have proven to be new to science. However, the most recent comprehensive treatment of the *Diplommatina* from Myanmar dates to the early 20^th^ century, published by Gude (1921). Although several new diplommatinids have recently been described from Myanmar amber fossil dating to the early to mid-Cretaceous (e.g., Yu et al. 2018, 2021; Hirano et al. 2019; Bichain et al. 2025), these findings do not contribute to the understanding of the extant fauna. The limited knowledge of the recent diplommatinids from Myanmar is largely due to the fact that faunistic studies in the country were historically confined to the British colonial period, resulting in a substantial gap in modern systematic research on this genus.

Under the project ‘Conserving Myanmar’s Karst Biodiversity’, jointly implemented by Fauna & Flora International (FFI) and the Forestry Department of Myanmar, the authors participated in fieldwork conducted in Myanmar between 2015 and 2017. A detailed examination of the external shell morphology of the collected material revealed six known taxa and two undescribed taxa belonging to the genera *Diplommatina* and *Pagodapalaina* Chen, 2025. Most *Diplommatina* species from Myanmar were originally described by British naturalists before the late 1800s, and their type specimens are primarily housed in the Natural History Museum, London. As type specimens are critical for facilitating taxonomic revisions and validating taxa (Winston 1999; Xie et al. 2025), their study is essential for establishing a reliable taxonomic framework. The present study aims to narrow the existing knowledge gap concerning *Diplommatina* s.l. in Myanmar through a comprehensive taxonomic revision, in which we describe two new species and redescribe six known species based on newly collected material from multiple localities. Furthermore, we update the species account of *Diplommatina* s.l. in Myanmar and compile information on all available type specimens, providing a valuable reference for future taxonomic and systematic studies in the Indochina region.

## Materials and methods

**Figure 1.**
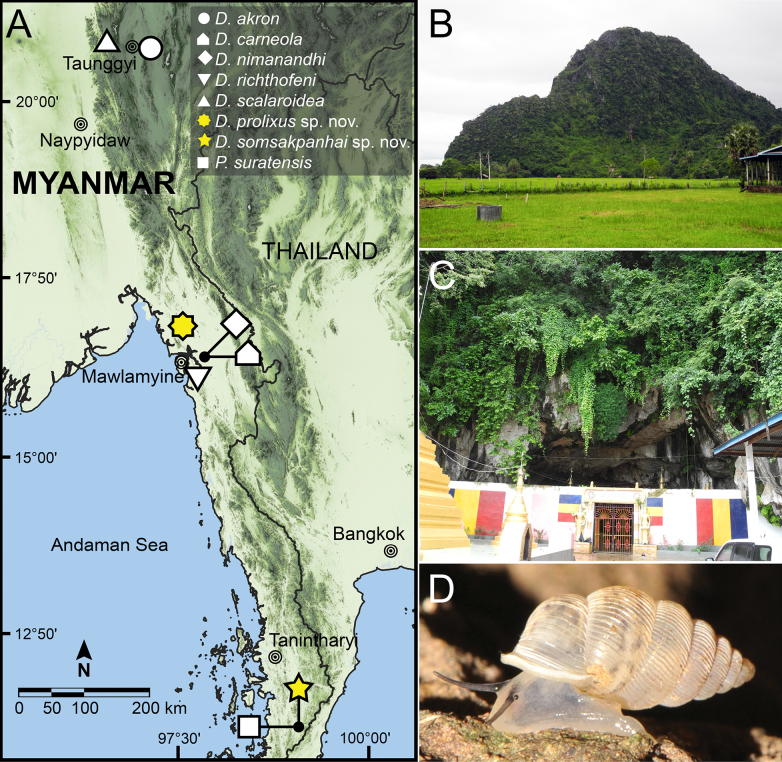
**A**. Geographic distribution map of *Diplommatina* and *Pagodapalaina* species in Myanmar based on the specimens examined in this study. White symbols indicate previously described species. Yellow symbols indicate new species described herein; **B**. Western part of Damathat Hill photographed from the northern side; **C**. Entrance of the famous Damathat Cave, which has been designated as the type locality for several species. The cave is currently occupied by a Buddhist temple; **D**. *Diplommatina akron* Panha & Burch, 1998, specimen CUMZ 15451 from Aye Say Tee Cave, Shan State.

A malacological survey in Myanmar was conducted between 2015 and 2017 through the collaboration of Fauna & Flora International, the Department of Forest Resource Management, Ministry of Natural Resources and Environmental Conservation of Myanmar, and Chulalongkorn University, Thailand. Specimens were collected by direct visual searching and hand collecting at several accessible localities across the Mandalay Region, Shan State, Mon State, Kayin State, and Tanintharyi Region (Fig. 1A–C). Prior to preservation, live specimens were photographed *in situ* to document their colouration and appearance in life (Fig. 1D). The snails were then euthanised following the standard two-step protocol recommended by the American Veterinary Medical Association (2020) and subsequently preserved in 70% ethanol (v/v). All specimens were identified based on shell characters, following the diagnostic characters provided in the literature (Blanford 1862, 1863, 1865; Theobald 1870; Stoliczka 1871; Theobald and Stoliczka 1872; Godwin-Austen 1884, 1886; Gude 1921; Panha and Burch 2005; Budha et al. 2017), and were compared with type specimens when available.

For description, shells were cleaned, examined under an Olympus SZX2-TR30 stereoscopic light microscope, and imaged using a JEOL JSM-6610 LV scanning electron microscope (SEM). Shell morphology, including overall shape, sculpture, striation, and the embryonic shell sculpture, was described, and measurements were taken under the light microscope following the criteria outlined by Neubert and Bouchet (2015), Greke (2017) and Nurinsiyah and Hausdorf (2017). Diagnoses and morphological descriptions are provided for all *Diplommatina* s.l. species recorded during the present survey in Myanmar. Brief diagnostic characters are also provided for species previously recorded from Myanmar, based primarily on type specimens and authenticated material.

To examine the internal folds and lamellae, micro–X-ray computed tomography (micro-CT) scans were conducted at the Scientific and Technological Research Equipment Center (STREC), Chulalongkorn University. Samples were mounted on a rotating stage and scanned using a high-resolution microtomography system (Bruker SkyScan 1273, Bruker microCT, Belgium). Scanning was performed at 40 kV and 50 µA with a rotation step of 0.3°, using a distortion-free flat-panel detector (3072 × 3072 pixels). Projection images were acquired over 360° and subsequently corrected for beam-hardening and ring artifacts using the manufacturer’s standard algorithms. Reconstructed cross-sectional images were generated with NRecon version 2.2.0.6 (Bruker microCT), producing isotropic voxel sizes of 4.24 μm. Segmentation and 3D visualisation of internal structures were performed using CTvox version 3.3 (Bruker microCT). The micro-CT data files (.vxm) for the new and redescribed species are provided in the Suppl. material 1.

### Institutional abbreviations

The type materials and examined specimens from museum collections are as follows:

**CUMZ** Chulalongkorn University, Museum of Zoology, Bangkok

**NHM** The Natural History Museum, London (NHMUK, when citing specimen lots deposited therein)

**NMW** National Museum of Wales, Cardiff

**SMF** Senckenberg Forschungsinstitut und Naturmuseum, Frankfurt am Main

### Photograph credits

Photographs of the type specimens and specimens from the museum collections are credited to each respective museum.

### Systematic descriptions

#### Superfamily Cyclophoroidea Gray, 1847

**Family Diplommatinidae Pfeiffer, 1856**

##### Genus Diplommatina

Taxon classificationAnimaliaArchitaenioglossaDiplommatinidae

Benson, 1849

61FF5028-ABD9-591C-ACAB-25539F119C3E

Diplommatina Benson, 1849: 193, 194. Godwin-Austen 1886: 166–169. Kobelt 1902: 423. Gude 1921: 301. Thiele 1929: 110, 111. Vermeulen 1993: 5. Egorov 2013: 17.

###### Type species.

*Bulimus folliculus* Pfeiffer, 1846, by subsequent designation of Nevill (1878: 284).

###### Remarks.

To date, more than 420 species and subspecies have been documented within the genus (Kobelt 1902; MolluscaBase 2025). Phylogenetic evidence indicates that the genus is polyphyletic (Rundell 2008; Webster et al. 2012; Köhler 2023). Consequently, several previously recognised subgenera have been elevated to generic rank, including *Moussonia* Semper, 1865 and *Gastroptychia* Kobelt & Möllendorff, 1900 (Neubert and Bouchet 2015; Chen and Ablett 2024). In addition, several new genera have been proposed, such as *Pagodapalaina* Chen, 2025, *Sohtsuia* Chen, 2023, and *Sinoarinia* Chen & Wu, 2020 (Chen and Wu 2020; Chen et al. 2023, 2025).

To date, 26 species of *Diplommatina* s.l. have been reported from Myanmar. Of these, 15 species have type localities within Myanmar, whereas the remaining ten species were originally described from neighbouring countries but were listed as occurring in ‘Burma’ or ‘Burmah’ (Gude 1921; Theobald and Stoliczka 1872). In addition, three species, *Pagodapalaina suratensis* (Panha & Burch, 1998), *D. akron* Panha & Burch, 1998, and *D. nimanandhi* Panha, Kanchanasaka & Burch, 2002, previously known only from Thailand, are recorded from Myanmar for the first time in the present study.

##### 1 Diplommatina scalaroidea

Taxon classificationAnimaliaArchitaenioglossaDiplommatinidae

Theobald, 1870

7188197A-CAB5-5B3A-BAEB-BE8D1940B550

Figs 2, 3 A

Diplommatina scalaroidea Theobald, 1870: 399, pl. 18, fig. 5. Type locality: Mandalay, regno Burmanico. Pfeiffer 1875: 80. Hanley and Theobald 1876: 56, pl. 141, fig. 10. Theobald 1876: 42. Nevill 1878: 285. Gude 1921: 333.
Diplommatina (Eudiplommatina) scalaroidea —Kobelt and Möllendorff 1898: 138.
Diplommatina (Diplommatina) scalaroidea —Kobelt 1902: 445.

###### Type material examined.

The type series could not be located in the Natural History Museum, London, nor the National Museum of Wales, Cardiff (T. White, B. Rowson, and J. Gallichan, pers. comm.).

###### Other material.

**Figure 2.**
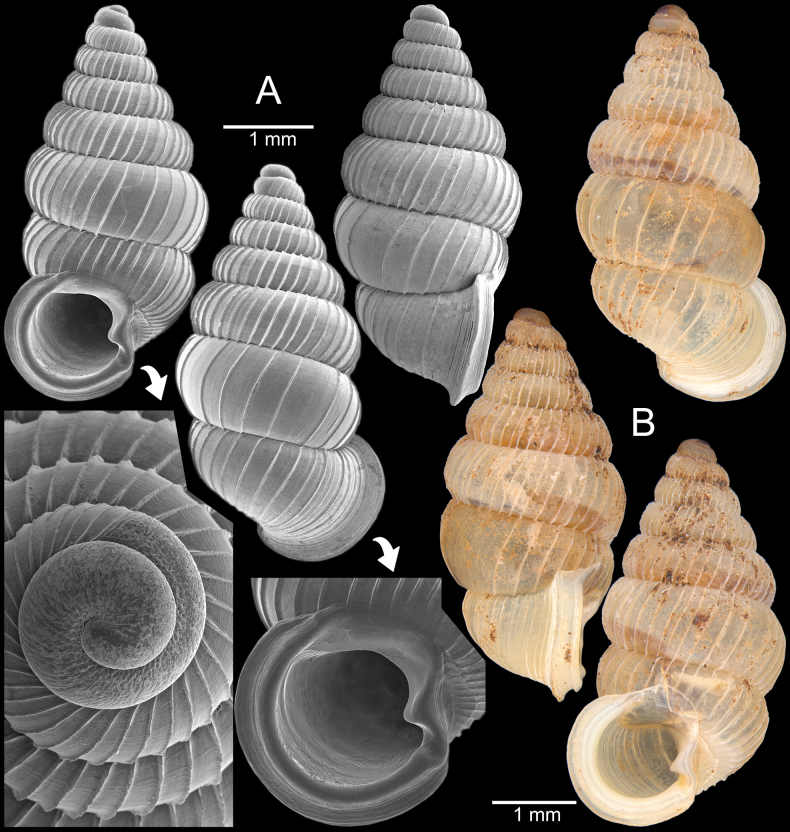
*Diplommatina scalaroidea* Theobald, 1870 from Pyiyaung, Mandalay Region, Myanmar. **A**. Specimen CUMZ 15442, insets show the protoconch sculpture, and aperture with columellaris; **B**. Specimen CUMZ 15443.

**Figure 3.**
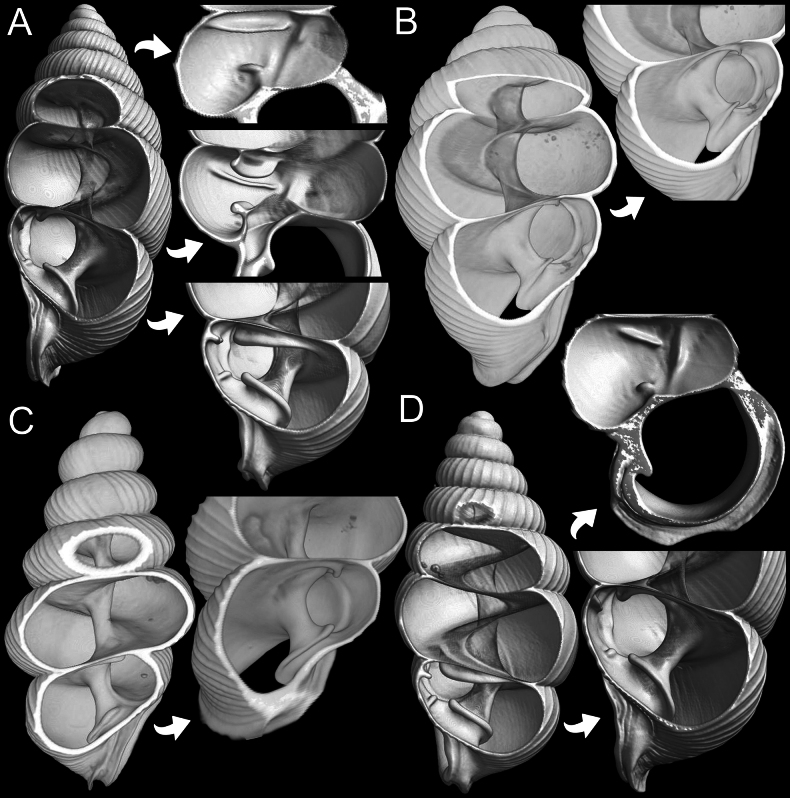
Micro-CT image showing shell barrier. **A**. *Diplommatina scalaroidea* Theobald, 1870, specimen CUMZ 15443; **B**. *Diplommatina carneola* Stoliczka, 1871, specimen CUMZ 15446; **C**. *Diplommatina richthofeni* Theobald & Stoliczka, 1872, specimen CUMZ 15449; **D**. *Diplommatina akron* Panha & Burch, 1998, specimen CUMZ 15452.

Myanmar • CUMZ 15442 (2 shells; Fig. 2A), CUMZ 15443 (1 shell; Figs 2B, 3A) and CUMZ 15444 (12 shells + 3 juveniles) from Pyiyaung city, Mandalay Region; 20°50'47.5"N, 96°24'12.4"E; P. Tongkerd and S. Panha leg.

###### Description.

Shell sinistral, ovoid-fusiform, pale yellowish to whitish colour. Shell height 4.6–5.2 mm; shell width 2.3–2.6 mm. Whorls ~7–8, convex; spire conical; suture wide and deep. Protoconch ~1¾ whorl, with fine growth lines, wrinkles, and roundish malleated pits. Teleoconch with strong, nearly regularly spaced radial ribs; penultimate whorl with widest spacing ribs, ~3–4 ribs/mm; last whorl before aperture with ~5–6 ribs/mm. Spiral striations very thin, fine and appear from teleoconch to about fourth whorl. Penultimate whorl wider than last whorl. Constriction externally inconspicuous; internal constriction marked by distinct vertical ridge. Aperture circular, with four barriers: parietalis very strong and prominent ridge; two horizontal palatalis situated beyond constriction–upper palatalis elongate with prominent ridge, lower palatalis short and strong; columellaris distinctly prominent ridge, continuous with peristome, and slightly deflected downward toward palatal wall (Fig. 3A). Peristome broadly expanded, double; outer peristome broader than inner peristome. Lip thickened, multi-layered; parietal callus thin, expanded. Columellar angulate, with distinct sinulus below. Umbilicus closed.

###### Distribution.

This species was originally described from the Mandalay Region. During our surveys across multiple localities in the Mandalay Region and Shan State, the species was found exclusively on limestone karsts near Pyiyaung City, suggesting that its distribution is likely restricted to the Mandalay Region (Fig. 1A).

###### Remarks.

Theobald (1870: 398) described four *Diplommatina* species, namely *D. affinis* Theobald, 1870, *D. pupaeformis* Theobald, 1870, *D. salwiniana* Theobald, 1870 and *D. scalaroidea*, consecutively in the same publication but without clearly indicating the number of specimens examined, illustrations, or specifying the type localities. However, in the introduction, Theobald (1870) mentioned that the specimens were obtained from Mr. Fedden, who had collected them after returning from the Upper Salwin. The type series of these four species could not be located in the NHM, London, nor the NMW, Cardiff collections, except for *D. pupaeformis* (see under *D. pupaeformis*; T. White, B. Rowson, and J. Gallichan, pers. comm.).

Although the type specimen of *D. scalaroidea* is not available, the diagnostic characters given in the original description and the type locality correspond well with the specimens examined in this study. This species is readily distinguished from all other congeners by its sinistral shell coiling, whereas all other species possess dextral shells. The most closely related species in Myanmar is *D. exilis* Blanford, 1863 from Ava, which differs in having a dextral shell and more narrowly spaced radial ribs (Hanley and Theobald 1876).

##### 2 Diplommatina carneola

Taxon classificationAnimaliaArchitaenioglossaDiplommatinidae

Stoliczka, 1871

1984A848-5480-5010-BA50-9F2C8319F54D

Figs 3B, 4, 5

Diplommatina carneola Stoliczka, 1871: 152, 153, pl. 6, fig. 3. Type locality: Damotha, prope Moulmein. Pfeiffer 1875: 76, 77. Hanley and Theobald 1876: 55, pl. 140, fig. 4. Theobald 1876: 42. Godwin-Austen 1884: pl. 49, figs 8, 8a. Godwin-Austen 1886: 182, 183. Gude 1921: 346.
Diplommatina *carreola* [sic]—Nevill 1878: 284.
Diplommatina (Sinica) carneola —Kobelt and Möllendorff 1898: 140. Kobelt 1902: 459.

###### Type material examined.

**Figure 4.**
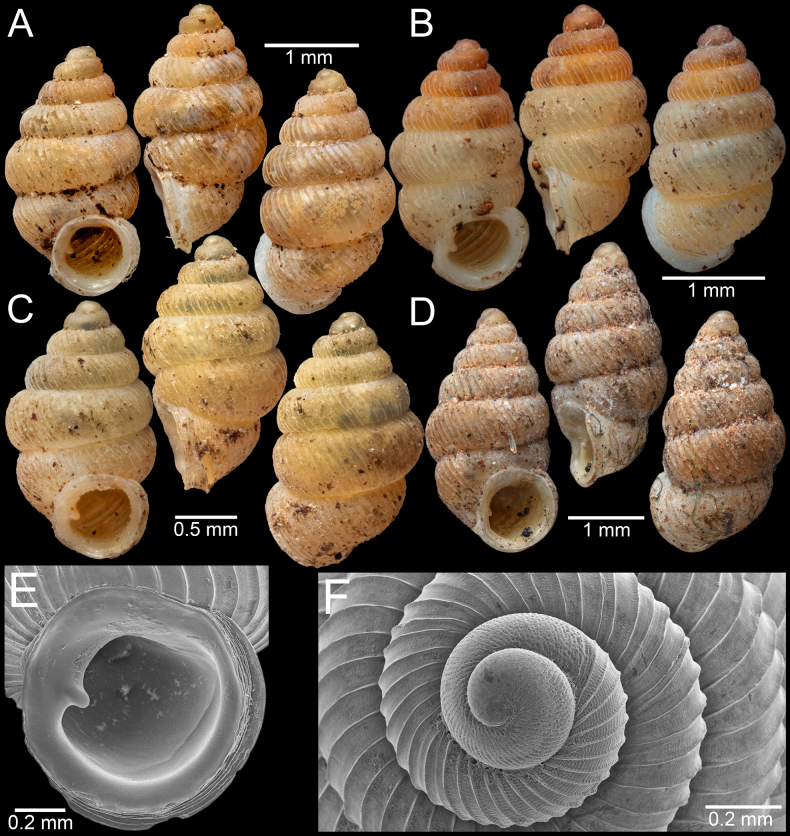
*Diplommatina carneola* Stoliczka, 1871. **A**. Syntype NHMUK 1871.9.23.33 from Moulmein, Tenasserim, Burma; **B**. Specimen NHMUK 1888.12.4.171–174 from Damathat, Moulmein; **C**. Syntype NHMUK 1903.7.1.2279 from Moulmein, Tenasserim, Burma; **D**. Specimen NHMUK 1903.7.1.2199 from Moulmein, Burma; **E, F**. Specimens CUMZ 15445 from Damathat Cave, Mon State, Myanmar: **E**. Enlarged view of the aperture with columellaris; **F**. Enlarged view showing protoconch sculpture.

Myanmar • ***Syntypes***. NHMUK 1871.9.23.33 (2 shells; Fig. 4A) from Moulmein, Tenasserim, Burma; Damon ex. Stoliczka coll. • ***Syntype***. NHMUK 1903.7.1.2279 (1 shell; Fig. 4C) from Moulmein, Tenasserim, Burma; Stoliczka coll.

###### Other material.

**Figure 5.**
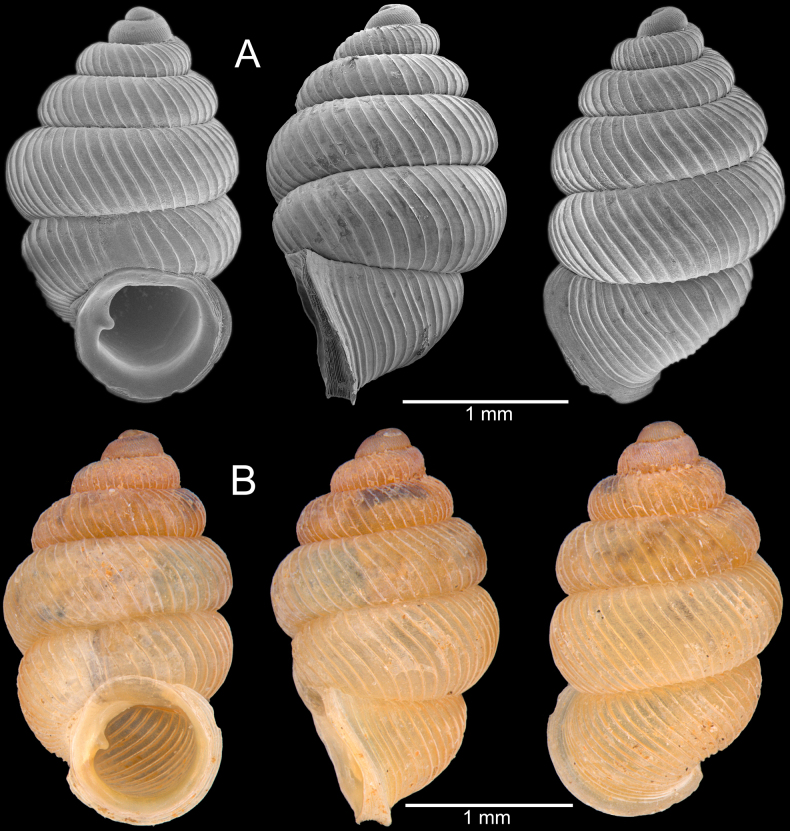
*Diplommatina carneola* Stoliczka, 1871 from Damathat Cave, Mon State, Myanmar. **A**. Specimen CUMZ 15445; **B**. Specimen CUMZ 15446.

Myanmar • NHMUK 1888.12.4.171–174 (4 shells; Fig. 4B) from Damathat, Moulmein; Theobald coll. • NHMUK 1903.7.1.2199 (1 shell; Fig. 4D); from Moulmein, Burma; Godwin-Austen coll. • NHMUK 20240011 (1 shell); from Moulmein, Burma; Sykes coll. • NHMUK 20240012 (1 shell); from Moulmein, Burma; Salisbury coll. • CUMZ 15445 (2 shells; Figs 4E, 4F, 5A), CUMZ 15446 (5 shells; Figs 3B, 5B) and CUMZ 15447 (72 shells) from Damathat Cave, Mawlamyine City, Mon State; 16°30'23.0"N, 97°48'36.3"E; P. Tongkerd and S. Panha leg.

###### Description.

Shell dextral, ovoid-fusiform, pale yellowish orange to yellowish. Shell height 2.0–2.3 mm; shell width 1.3–1.4 mm. Whorls 5–6, well rounded; spire depressed conical; suture wide and deep. Protoconch ~1¾ whorls, with wrinkles and roundish malleated pits arranged in radial rows. Teleoconch with strong, regularly spaced radial ribs; ~5–6 ribs/0.5 mm on both penultimate and last whorls before aperture. Last whorl slightly narrower than penultimate whorl. Spiral striations very fine and weak, and appearing throughout. Penultimate whorl wider than last whorl. Constriction externally inconspicuous; internal constriction with distinct vertical ridge. Aperture circular, with three barriers: parietalis weak, low ridge; palatalis short, slightly prominent, and situated close to internal constriction ridge; columellaris prominent, continuous with peristome, and slightly deflected downward toward palatal wall (Fig. 3B). Peristome expanded, double; outer peristome slightly broader than inner peristome. Lip thickened, multi-layered; parietal callus thick or thin and weakly expanded. Columella short and curved. Umbilicus closed.

###### Distribution.

The species is currently known from its type locality ‘Damotha, near Moulmein’, which corresponds to the modern locality name Damathat Cave (16°30'21.8"N, 97°49'11.5"E), Mawlamyine, Mon State, Myanmar (Fig. 1A–C).

###### Remarks.

Among the *Diplommatina* species occurring in Mon and Kayin states, Myanmar, *D. carneola* is similar to *D. polypleuris* Benson, 1857 in having ovoid-fusiform shell shape (Kobelt 1902) but *D. carneola* presents very closely arranged transverse costae.

##### 3 Diplommatina richthofeni

Taxon classificationAnimaliaArchitaenioglossaDiplommatinidae

Theobald & Stoliczka, 1872

73C70A59-278F-5FCD-AAE3-3578A255CEBF

Figs 3C, 6, 7A

Diplommatina richthofeni Theobald & Stoliczka, 1872: 331, 332, pl. 11, fig. 4. Type locality: Prope Moulmain [Mawlamyine, Myanmar]. Pfeiffer 1875: 78, 79. Hanley and Theobald 1876: 56, pl. 141, figs 7, 8. Theobald 1876: 42. Nevill 1878: 285. Gude 1921: 331.
Diplommatina (Eudiplommatina) richthofeni —Kobelt and Möllendorff 1898: 137.
Diplommatina (Diplommatina) richthofeni —Kobelt 1902: 443.

###### Type material examined.

Myanmar • ***Holotype***. NHMUK 1888.12.4.162 (Fig. 7A) from Moulmein; Theobald coll.

**Figure 6.**
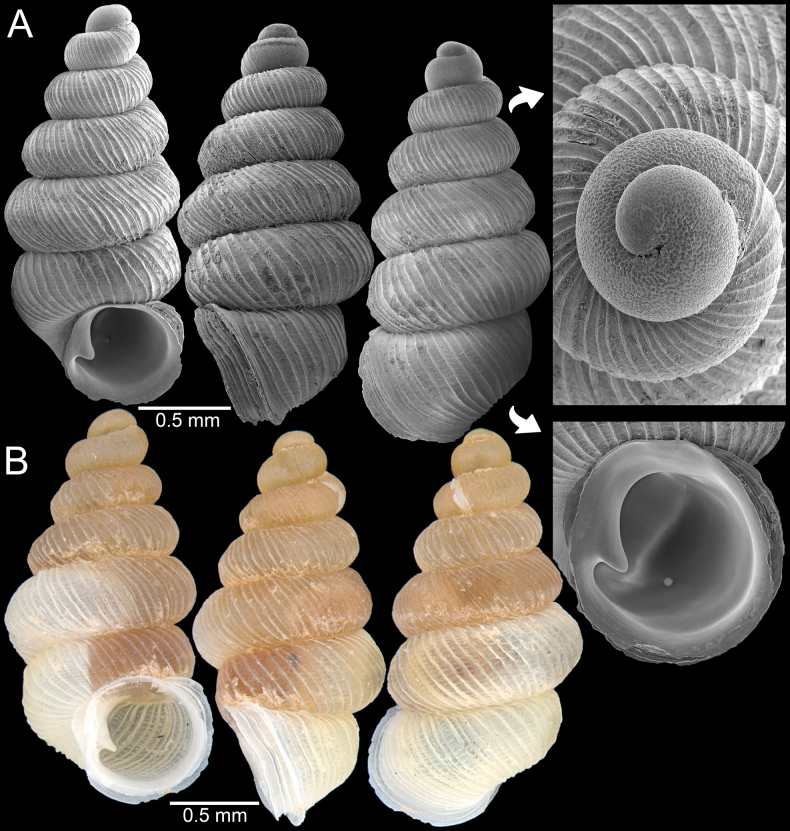
*Diplommatina richthofeni* Theobald & Stoliczka, 1872 from Naga Mauk Cave, Mon State, Myanmar. **A**. Specimen CUMZ 15448, insets show the protoconch sculpture, and aperture with columellaris; **B**. Specimen CUMZ 15449.

**Figure 7.**
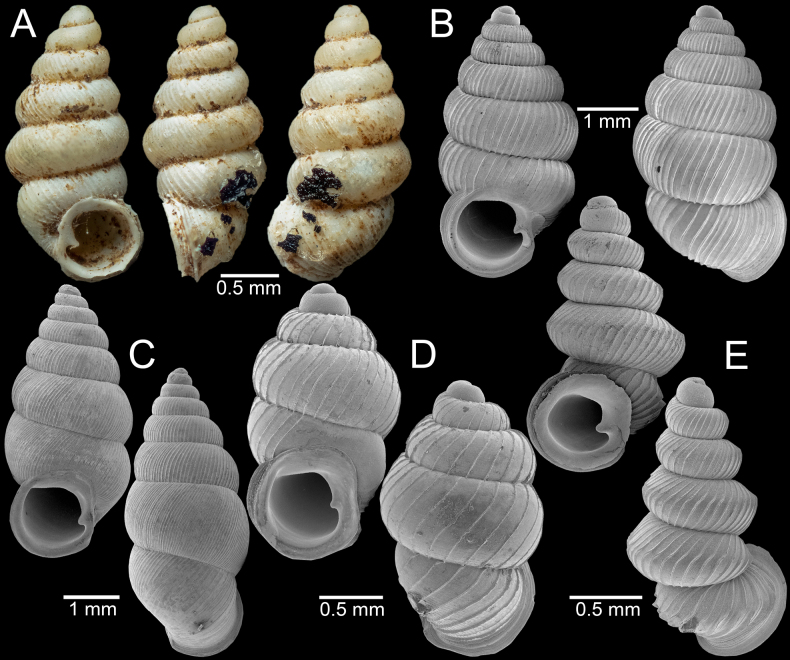
**A**. *Diplommatina richthofeni* Theobald & Stoliczka, 1872, holotype NHMUK 1888.12.4.162 from Moulmein; **B**. *Diplommatina akron* Panha & Burch, 1998, holotype CUMZ 15398 (former Di 022) from Chiang Mai Province, Thailand; **C**. *Diplommatina inthanon* Panha & Burch, 2001, holotype CUMZ 15394 (former IDi-087) from Chiang Mai Province, Thailand; **D**. *Diplommatina siriphumi* Panha & Burch, 2001, holotype CUMZ 15372 (former IDi-090) from Chiang Mai Province, Thailand; **E**. *Pagodapalaina suratensis* (Panha & Burch, 1998), holotype CUMZ 15370 (former Di 028) from Surathani Province, Thailand.

###### Other material.

Myanmar • CUMZ 15448 (1 shell; Fig. 6A), CUMZ 15449 (1 shell; Figs 3C, 6B) and CUMZ 15450 (85 shells) from Naga Mauk Cave, Mawlamyine City, Mon State; 16°18'58.4"N, 97°42'23.0"E; P. Tongkerd and S. Panha leg.

###### Description.

Shell small, dextral, spindle-shaped, pale whitish to pale yellowish. Shell height 2.1–2.5 mm; shell width 0.8–1.1 mm. Whorls ~6, slightly angular-rounded; spire conical; suture wide and deep. Protoconch ~1¾ whorl, with prominent round malleated pits throughout. Teleoconch with strong, irregularly spaced, and sinuous radial ribs; ~10–11 ribs/0.5 mm on penultimate whorl, ~8–9 ribs/0.5 mm on last whorl towards aperture. Spiral striations very fine, appearing throughout from teleoconch to last whorl near aperture. Penultimate whorl slightly wider than last whorl. Constriction externally inconspicuous; internal constriction marked with low vertical ridge. Aperture circular, with two barriers: parietalis weak, low ridge; palatalis absent; columellaris prominent ridge, continuous with peristome, and slightly deflected downward toward palatal wall (Fig. 3C). Peristome expanded; outer peristome broader than inner peristome. Lip multi-layered; parietal callus thin, expanded. Columella short and curved. Umbilicus sealed.

###### Distribution.

This species is currently known only from Naga Mauk Cave, Mawlamyine City, Mon State, Myanmar (Fig. 1A). The original locality was recorded as ‘Moulmain’.

###### Remarks.

Theobald and Stoliczka (1872) clearly stated in the original description that only a single specimen was obtained from Farm Caves.

##### 4 Diplommatina akron

Taxon classificationAnimaliaArchitaenioglossaDiplommatinidae

Panha & Burch, 1998

5AB94CB3-8C3B-51AF-A45B-C01E1EEF260F

Figs 1D, 3D, 7B, 8

Diplommatina akron Panha & Burch, 1998[1996]: 52–54, fig. 3. Type locality: Doichiang Dao Wildlife Sanctuary, Chiangmai, Thailand. Panha and Burch 2005: 19, fig. 19. BEDO 2017: 75. Jirapatrasilp et al. 2023: 26.

###### Type material examined.

Thailand • ***Holotype***. CUMZ 15398 (former Di-022; Fig. 7B) from Doichiang Dao Wildlife Sanctuary, Chiang Mai; Panha and Burch coll. • ***Paratypes***. CUMZ 15399 (former Di-023; 16 shells); same data as for holotype.

**Figure 8.**
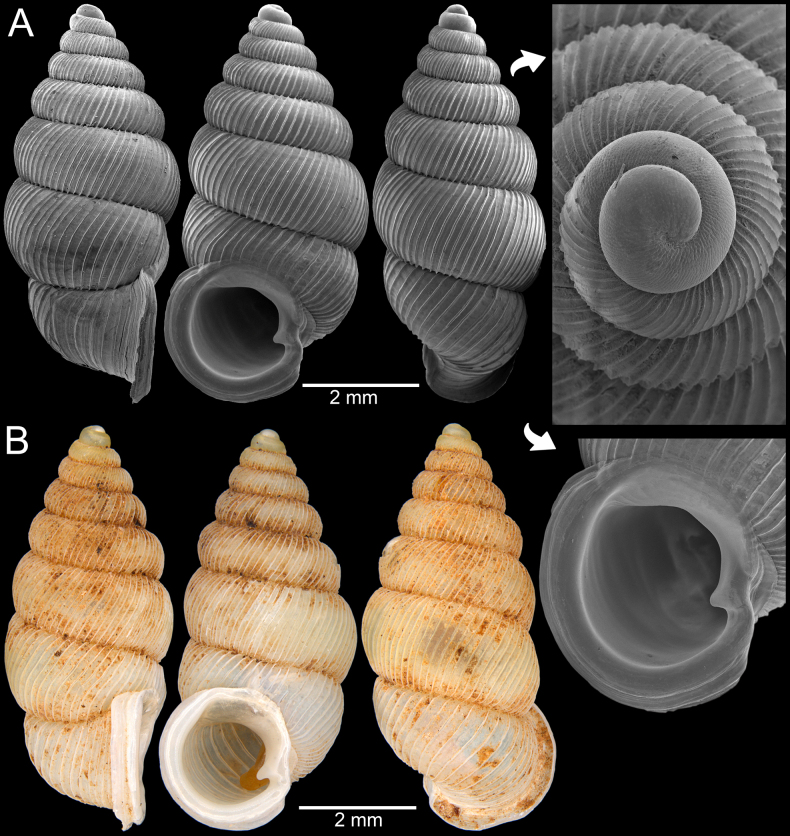
*Diplommatina akron* Panha & Burch, 1998 from Taunggyi, Shan State, Myanmar. **A**. Specimen CUMZ 15451, insets show the protoconch sculpture, and aperture with columellaris; **B**. Specimen CUMZ 15452.

###### Other material.

Myanmar • CUMZ 15451 (1 shell; Figs 1D, 8A) and CUMZ 15452 (1 shell; Figs 3D, 8B) from Aye Say Tee Cave, Taunggyi City, Shan State; 20°47'29.5"N, 97°03'01.6"E; P. Tongkerd and S. Panha leg.

###### Description.

Shell sinistral, fusiform to nearly conical, relatively large, and ivory white or transparent yellow. Shell height 4.9–5.8 mm; shell width 2.6–3.1 mm. Whorls ~7–8, rounded; spire conical; suture wide, deep. Protoconch ~1¾ whorl, nearly smooth, with weak wrinkles and coalescing round malleated pits. Teleoconch with delicate costae, later becoming stronger, regularly spaced radial ribs; ~8–9 ribs/mm on penultimate whorl towards aperture. Spiral striations very weak, restricted to early whorls (seen under SEM). Penultimate whorl slightly wider than last whorl. Constriction externally inconspicuous; internal constriction marked with distinct vertical ridge. Aperture circular, with four barriers: parietalis very strong, prominent, tall ridge; two horizontal palatalis situated beyond constriction–upper palatalis elongate with prominent ridge, and lower palatalis short, prominent ridge; columellaris distinctly prominent ridge, continuous with peristome, and slightly deflected downward toward palatal wall (Fig. 3D). Peristome broadly expanded, double; outer peristome slightly broader than inner peristome. Lip thickened, multi-layered; parietal callus thickened, expanded. Columella angulate, with sinulus below. Umbilicus closed.

###### Distribution.

This species was originally described from northern Thailand and is also currently known from Shan State, Myanmar. In the present surveys, it was recorded from a limestone outcrop in Taunggyi, Shan State, at an elevation of 1,583 m (Fig. 1A). However, its occurrence in other parts of Shan State cannot be ruled out.

###### Remarks.

Although this species shares a sinistral and fusiform shell shape with *D. pupaeformis* from Shan State, Myanmar, and *D. inthanon* Panha & Burch, 2001 from Chiang Mai Province, Thailand, several diagnostic characters can be used to differentiate it from these congeners. For comparison, *D. akron* possesses a fusiform shell with strong, widely spaced radial ribs and a robust, prominent columellaris. In contrast, *D. pupaeformis* has an almost pupilliform shell, more narrowly spaced radial ribs, and a weaker columellaris. Additionally, *D. inthanon* (Fig. 7C) can be distinguished by its fine, thin, and densely arranged radial ribs (Theobald 1870; Panha and Burch 2001, 2005).

Living animal pale white in colour, with white tentacles (Fig. 1D).

##### 5 Diplommatina nimanandhi

Taxon classificationAnimaliaArchitaenioglossaDiplommatinidae

Panha, Kanchanasaka & Burch, 2002

599B3DC0-F67E-59FE-AA75-A0EAF8C92159

Figs 9, 10, 11 A

Diplommatina nimanandhi Panha, Kanchanasaka & Burch, 2002[1998]: 159–163, fig. 4. Type locality: Banpangcam Cave, Mae Hongson Province, Thailand. Panha and Burch 2005: 28, fig. 30. BEDO 2017: 81. Jirapatrasilp et al. 2023: 26.

###### Type material examined.

**Figure 9.**
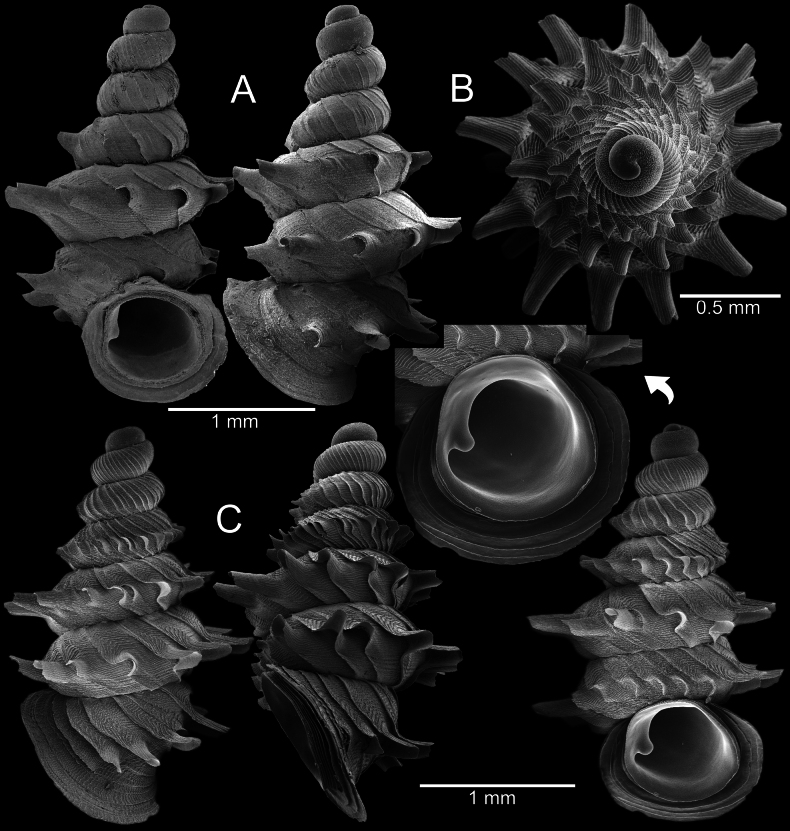
*Diplommatina nimanandhi* Panha, Kanchanasaka & Burch, 2002. **A**. Holotype CUMZ 15379 (former Di-037) from Maehongson Province, Thailand; **B, C**. Specimen CUMZ 15453 from Mawlamyine City, Mon State, Myanmar, inset show aperture with columellaris.

Thailand • ***Holotype***. CUMZ 15379 (former Di-037; Fig. 9A) ex. from Banpangcam Cave, Mae Hongson Province; Panha coll. • ***Paratypes***. CUMZ 15380 (former Di-029; 14 shells); same data as for holotype.

###### Other material.

**Figure 10.**
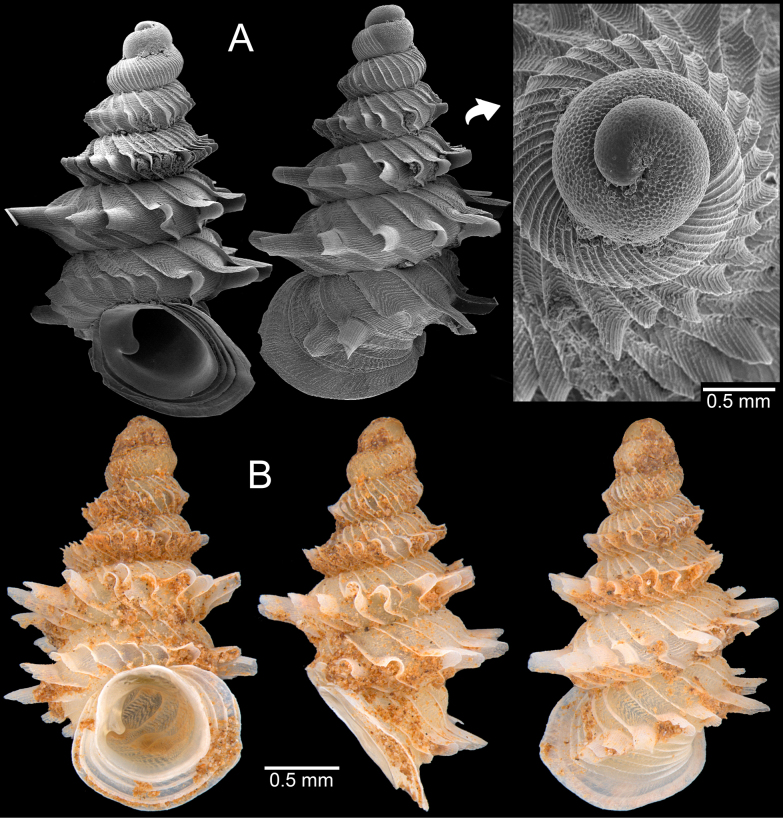
*Diplommatina nimanandhi* Panha, Kanchanasaka & Burch, 2002 from Mawlamyine City, Mon State, Myanmar. **A**. Specimen CUMZ 15453, inset show the protoconch sculpture; **B**. Specimen CUMZ 15454.

**Figure 11.**
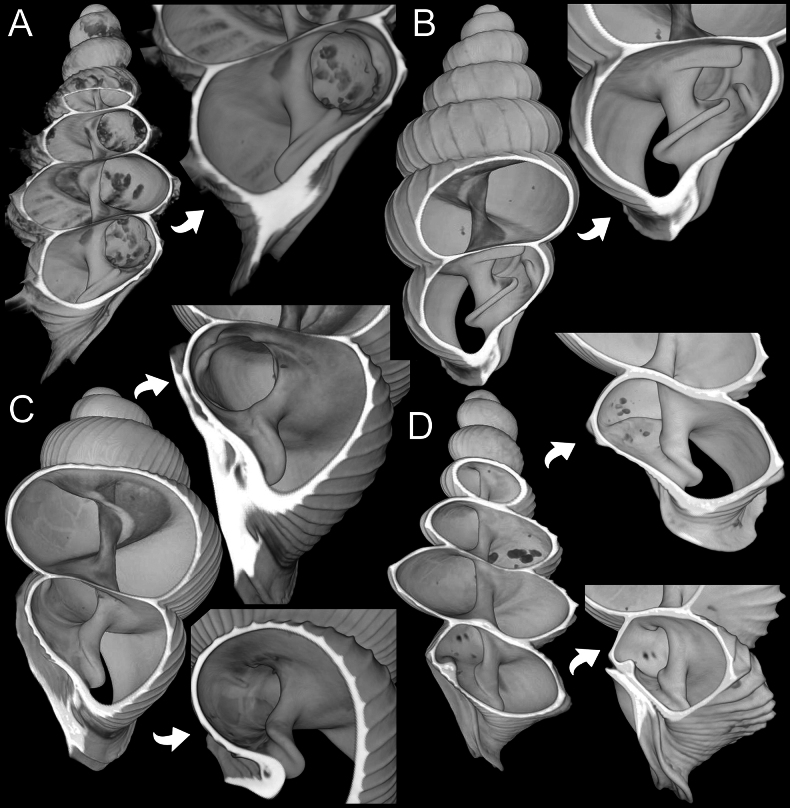
Micro-CT image showing shell barrier. **A**. *Diplommatina nimanandhi* Panha, Kanchanasaka & Burch, 2002, specimen CUMZ 15454 from Mon State, Myanmar; **B**. *Diplommatina prolixa* sp. nov., holotype CUMZ 15458 from Kayin State, Myanmar; **C**. *Diplommatina somsakpanhai* sp. nov., paratype CUMZ 15461 from Tanintharyi Region, Myanmar; **D**. *Pagodapalaina suratensis* (Panha & Burch, 1998), specimen CUMZ 15464 from Tanintharyi Region, Myanmar.

Myanmar • CUMZ 15453 (4 shells; Figs 9B, 9C, 10A), CUMZ 15454 (5 shells; Figs 10B, 11A) and CUMZ 15455 (69 shells + 8 juveniles) from Damathat Cave, Mawlamyine City, Mon State; 16°30'23.0"N, 97°48'36.3"E; P. Tongkerd and S. Panha leg.

###### Description.

Shell dextral, conical fusiform, whitish to yellowish. Shell height 2.7–2.9 mm; shell width 0.8–1.3 mm. Whorls ~6–7, rounded; spire conical; suture wide and deep. Protoconch ~1¾ whorl, with prominent round malleated pits throughout. Teleoconch with strong, delicate radial ribs: about first two whorls’ ribs regularly low then about last three whorls ribs gradually becoming more widely spaced, more prominent, sinuous, and very tall with trough-like shape at angulation, thinner near suture. Ribs with 2–3 ribs/0.5 mm on penultimate whorl; 3–4 ribs/0.5 mm on last whorl towards aperture. Spiral striations very fine, prominent, and appearing from teleoconch to aperture. Last whorl slightly narrower than penultimate whorl. Constriction externally inconspicuous; internal constriction marked with vertical ridge. Aperture circular, with two barriers: parietalis weak, low ridge to knob-like; palatalis absent; columellaris distinctly prominent ridge, continuous with peristome, slightly deflected downward toward palatal wall (Fig. 11A). Peristome broadly expanded; outer peristome broader than inner peristome. Lip thickened, multi-layered; parietal callus thickened and expanded. Columella straight and thickened. Umbilicus closed.

###### Distribution.

Previously, this species was known only from its type locality in Mae Hong Son Province, Thailand (Panha et al. 2002). In the present study, despite surveys at multiple localities, the species was found exclusively in Damathat Cave, Myanmar (Fig. 1A).

###### Remarks.

Among the four species *D. angulata* Theobald & Stoliczka, 1872, *D. carneola*, *D. crispata* Stoliczka, 1871, and *D. exserta* Godwin-Austen, 1886, which were described from Damathat Cave, *D. nimanandhi* is readily distinguishable by its highly prominent, trough-shaped radial ribs. The most similar species is *D. crispata*, which exhibits angular whorls and dense, sinuous radial ribs (Kobelt 1902), whereas *D. nimanandhi* possesses more widely spaced, trough-shaped radial ribs.

##### 6 Diplommatina prolixa

Taxon classificationAnimaliaArchitaenioglossaDiplommatinidae

Tongkerd sp. nov.

918F5E99-8F6C-5051-AD3C-D8F3AB0156C8

https://zoobank.org/059DEB86-6890-4381-A26D-D6B5692967D4

Figs 11B, 12A

###### Type material.

**Figure 12.**
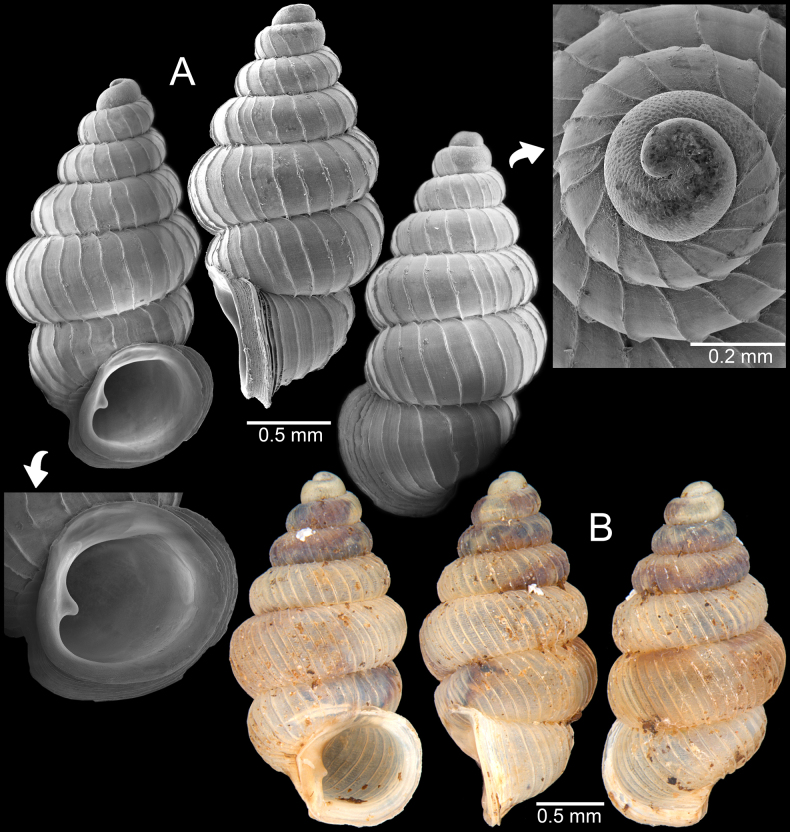
**A**. *Diplommatina prolixa* sp. nov., holotype CUMZ 15458 from Kayin State, Myanma, insets show the protoconch sculpture, and aperture with columellaris; **B**. *Diplommatina naiyanetri* Panha, 1998, paratype CUMZ 15383 (= Di 005) from Phatthalung Province, Thailand.

Myanmar • ***Holotype***. CUMZ 15458 (height 2.2 mm, width 1.2 mm; Figs 11B, 12A) from Rathye Pyan Cave, Hpa An City, Kayin State; 16°50'6.2"N, 97°34'14.5"E, 15 m a.s.l.; P. Tongkerd and S. Panha leg. • ***Paratype***. CUMZ 25459 (1 damaged shell); same data as for holotype.

###### Diagnosis.

Shell small, dextral, elongate-pyramidal, fusiform, ~6¼ convex whorls. Penultimate whorl widest. Shell with broadly spaced radial ribs, fine spiral striations throughout. Peristome double, expanded. Aperture rounded with three barriers: parietalis, palatalis and columellaris.

###### Description.

Shell dextral, elongate-pyramidal fusiform, whisper white or transparent yellow, apex orange. Shell height 2.0 and 2.2 mm; shell width 1.1 and 1.2 mm. Whorls 6¼, and broadly convex; spire elongate conical; suture wide and deep. Protoconch ~1½ whorls, with round malleated pits. Teleoconch with wide, regularly spaced radial ribs; ribs slightly sinuous, prominent near suture. Ribs with 3–4 ribs/0.5 mm on penultimate whorl, 4–5 ribs/0.5 mm on last whorl towards aperture. Spiral striations very weak, fine on only earlier whorls. Last whorl narrower than penultimate whorl. Constriction externally weakly conspicuous; internal constriction marked with distinct vertical ridge. Aperture circular with three barriers: parietalis very strong, prominent ridge; palatalis moderately long, strong, horizontal ridge; columellaris distinctly prominent, tall ridge, continuous with peristome, and slightly deflected downward toward palatal wall (Fig. 11B). Peristome broadly expanded, double; outer peristome broader than inner peristome. Lip thickened, multi-layered; parietal callus thick, expanded. Columella straight. Umbilicus closed.

###### Differential diagnosis.

*Diplommatina prolixa* sp. nov. is most similar in shell sculpture to *D. oligopleuris* Blanford, 1868 from India and *D. sperata* Blanford, 1862 from Rakhine State, Myanmar. However, it differs from *D. oligopleuris* in having an elongate fusiform shell, whereas *D. oligopleuris* has an ovate-fusiform shell. In addition, the new species can be distinguished from *D. sperata* by its more widely spaced radial ribs and a peristome edge that lacks protrusion, while *D. sperata* has more narrowly spaced radial ribs and a peristome with a protruding columellar edge.

For further comparison, *D. prolixa* sp. nov. can be distinguished from *D. naiyanetri* Panha, 1998 from southern Thailand by its wider spacing between the radial ribs, a rounded aperture, and without a pointed columellar edge. In contrast, *D. naiyanetri* possesses more narrowly spaced radial ribs, somewhat squarish aperture, and a clearly defined columellar notch. In addition, *D. naiyanetri* (Fig. 12B) was recorded from a locality approximately 1,040 km south of the type locality of this new species.

###### Distribution.

*Diplommatina prolixa* sp. nov. is currently known only from its type locality in Kayin State, Myanmar. This new species inhabits limestone cliff faces and rock crevices, with surrounding vegetation (Fig. 1A).

###### Etymology.

The specific name *prolixus* is a Latin word meaning wide or broad and alludes to broadly spaced radial ribs, which characterises this species.

###### Remarks.

The shell of *D. prolixa* sp. nov. is comparable to that of *D. exilis* from Mandalay Region, Myanmar (Kobelt 1902). However, the radial ribs of *D. exilis* are narrower than those of *D. prolixa* sp. nov.

##### 7 Diplommatina somsakpanhai

Taxon classificationAnimaliaArchitaenioglossaDiplommatinidae

Tongkerd sp. nov.

7FD3F5F3-7ADF-5BCA-8B30-41AD9596C857

https://zoobank.org/8D310ED2-667D-4632-A8B0-8567B08B400C

Figs 11C, 13

###### Type material.

**Figure 13.**
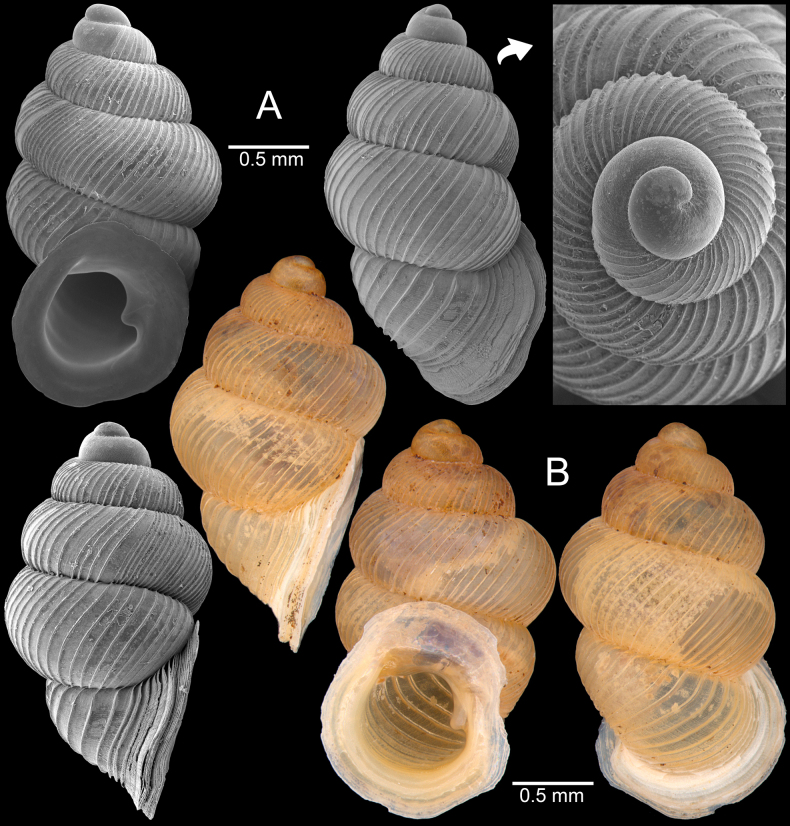
*Diplommatina somsakpanhai* sp. nov. from Tanintharyi Region, Myanmar. **A**. Holotype CUMZ 15460, inset show the aperture with columellaris; **B**. Paratype CUMZ 15461.

Myanmar • ***Holotype***. CUMZ 15460 (height 1.4 mm, width 2.2 mm; Fig. 13A) from Buddha Cave, Lenya City, Tanintharyi Region; 11°13'46.2"N, 99°10'34.3"E, 65 m a.s.l.; C. Sutcharit, R. Chanabun and R. Srisonchai leg. • ***Paratypes***. CUMZ 15461 (6 shells; Figs 11C, 13B) and CUMZ 15462 (30 shells + 20 juveniles); same data as for holotype. • NHMUK 20260057 (5 shells); same data as for holotype. • SMF (5 shells); same data as for holotype.

###### Diagnosis.

Shell small, sinistral, ovoid fusiform, ~5½ rounded whorls. Last whorl nearly same diameter as penultimate whorl. Shell with narrowly spaced radial ribs, fine spiral striations throughout. Peristome thickened, double, broadly expanded. Aperture rounded, with three barriers: parietalis, palatalis and columellaris.

###### Description.

Shell sinistral, ovoid-fusiform, pale orange or light yellowish. Shell height 1.3–1.5 mm; shell width 2.1–2.2 mm. Whorls ~5½, rounded; spire depressed conic; suture wide, deep. Protoconch ~1¾ whorls, nearly smooth to very weakly wrinkled or with roundish pits. Teleoconch with regular and narrowly spaced radial ribs; ribs spaced slightly wider near aperture; 10–11 ribs/0.5 mm on penultimate whorl, 7–8 ribs/0.5 mm on last whorl towards aperture. Spiral striations very fine, weakly appearing throughout. Last whorl slightly narrower than penultimate whorl. Constriction externally inconspicuous; internal constriction marked with indistinct vertical ridge. Aperture circular with three barriers: parietalis and palatalis weak, low ridges; columellaris distinctly prominent ridge, continuous with peristome, slightly deflected downward toward palatal wall (Fig. 11C). Peristome double, broadly expanded, and nearly or completely reaches suture; inner peristome broader than outer peristome. Lip thickened, multi-layered; parietal callus thickened, broadly expanded. Columella thickened and curved. Umbilicus sealed.

###### Differential diagnosis.

*Diplommatina somsakpanhai* sp. nov. can be distinguished from *D. siriphumi* Panha & Burch, 2001 from Chiang Mai Province, Thailand, by several diagnostic characters. The new species possesses more ovate whorls, a narrow space between radial ribs, a broadly expanded peristomal lip, and a large, distinct columellaris. In contrast, *D. siriphumi* (Fig. 7D) has a less ovate whorl, a wide space between radial ribs, a narrow peristomal lip, and a small, less prominent columellaris. In addition, *D. siriphumi* was recorded from a non-limestone habitat located approximately 700 km north of the type locality of this new species (Panha and Burch 2001, 2005).

Furthermore, the new species differs from *Pagodapalaina krabiensis* (Panha & Burch, 1998) from southern Thailand by its ovate-fusiform shell, ovate whorls, narrowly spaced radial ribs, and broadly expanded peristomal lip. In comparison, *P. krabiensis* possesses a fusiform shell with shouldered whorls, widely spaced radial ribs, and a narrowly expanded peristomal lip (Panha and Burch 1998, 2005).

###### Distribution.

*Diplommatina somsakpanhai* sp. nov. is currently known only from its type locality in the Tanintharyi Region, Myanmar. It inhabits limestone cliff faces and rock crevices, with surrounding vegetation (Fig. 1A).

###### Etymology.

The specific name *somsakpanhai* is derived from the full name of Professor Dr. Somsak Panha of Chulalongkorn University, in recognition of his remarkable contributions to the study of Southeast Asian land molluscs and biodiversity research in Thailand. This new species is named in his honour to celebrate the anniversary of his sixty-fifth birthday.

###### Remarks.

This new species is superficially similar to the type species of *Gastroptychia*, *G. adversa* (Adams & Adams, 1851), in having a sinistral, ovate-fusiform shell and a strongly expanded lip that is nearly attached to the suture (Adams and Adams 1851; Vermeulen 1993: 36, fig. 42). Although *Gastroptychia* has recently been resurrected as a valid genus, its diagnostic characters and distribution remain intermixed with the highly diverse genus *Diplommatina*, and the characters supporting its separation are still provisional (Egorov 2013; Chen and Ablett 2024). Current recognition of *Gastroptychia* is mainly based on molecular phylogenetic evidence (Webster et al. 2012), but this analysis did not include the type species. Therefore, in the absence of clear generic diagnostic characters, we provisionally place this new species in the widespread genus *Diplommatina*, pending a clearer definition of the diagnostic characteristics of *Gastroptychia*.

There are very few sinistral *Diplommatina* species that have been recorded from Myanmar, only five of 31 known species that occur in Myanmar, namely *D. salwiniana*, *D. scalaroidea*, *D. akron*, *P. suratensis*, and the new species described herein.

##### Genus Pagodapalaina

Taxon classificationAnimaliaArchitaenioglossaDiplommatinidae

Chen, 2025

A73011C5-B78A-5B75-B418-659D728C96C9

Pagodapalaina Chen in Chen et al. 2025: 390, 391.

###### Type species.

*Pagodapalaina hortulanica* Chen, 2025, by original designation.

###### Remarks.

The genus was recently separated from the more diverse *Diplommatina* s.l. based on differences in radular and shell morphology, as well as published molecular data (Webster et al. 2012; Boonmachai et al. 2023; Chen et al. 2025). It is characterised by a small, sinistral, conical to elongate-conical shell with angular whorls and prominent radial ribs that often form trough-like projections along the shell periphery. The genus currently comprises 17 species, which are mainly distributed in the Malay Peninsula and Sumatra, Indonesia. In Myanmar, the genus is recorded for the first time, as *P. suratensis* from the southern part of the Tanintharyi Region.

##### 8 Pagodapalaina suratensis

Taxon classificationAnimaliaArchitaenioglossaDiplommatinidae

(Panha & Burch, 1998)

FE717513-F89D-56CF-8963-226871607C17

Figs 7E, 11D, 14

Diplommatina suratensis Panha & Burch, 1998: 57, fig. 5. Type locality: Klongsang Wildlife Sanctuary, Surathani Province, Thailand. Panha and Burch 2005: 33, fig. 35. BEDO 2017: 83. Jirapatrasilp et al. 2023: 26.
Pagodapalaina suratensis —Chen et al. 2025: 398, 399, fig. 3h.

###### Type material examined.

Thailand • ***Holotype***. CUMZ 15370 (former Di 028; Fig. 7E) from Klongsang Wildlife Sanctuary, Surathani Province; Panha and Burch coll. • ***Paratypes***. CUMZ 15371 (former Di 029; 7 shells); same data as for holotype.

**Figure 14.**
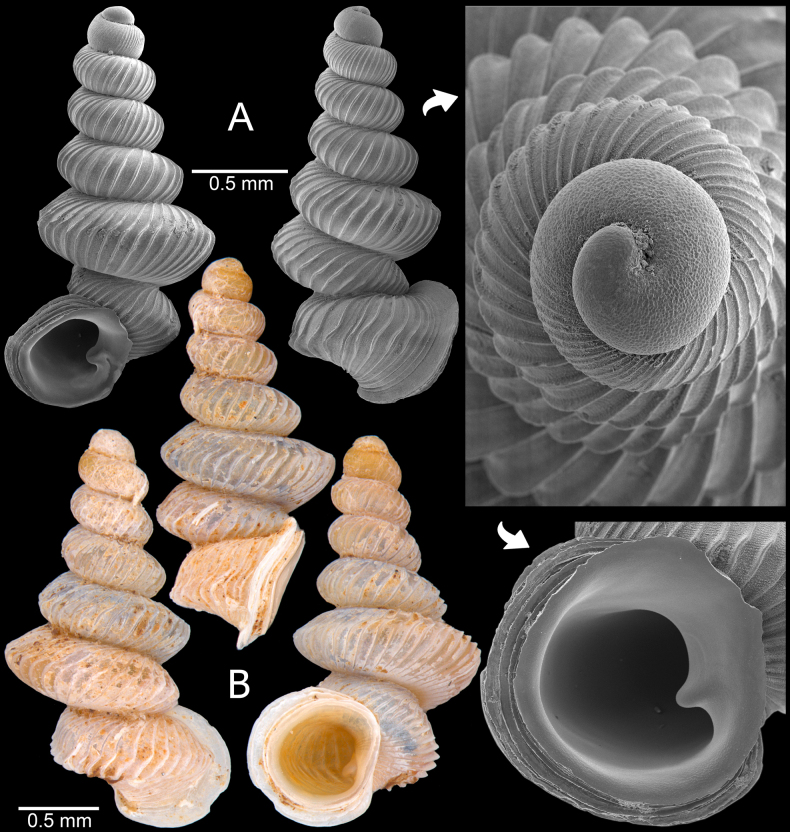
*Pagodapalaina suratensis* (Panha & Burch, 1998) from Tanintharyi Region, Myanmar. **A**. Specimen CUMZ 15463, insets show the protoconch sculpture, and aperture with columellaris; **B**. Specimen CUMZ 15464.

###### Other material.

Myanmar • CUMZ 15463 (1 shell; Fig. 14A), CUMZ 15464 (1 shell; Figs 11D, 14B), CUMZ 15465 (327 shells + 6 juveniles) from Buddha Cave, Lenya City, Tanintharyi Region; 11°13'46.2"N, 99°10'34.3"E; C. Sutcharit, R. Chanabun and R. Srisonchai leg.

###### Description.

Shell sinistral, turreted-fusiform, reddish to yellowish. Shell height 1.9–2.2 mm; shell width 0.9–1.2 mm. Whorls ~7, earlier whorls rounded, last three whorls angular; spire elongate conical; suture wide and very deep. Protoconch ~1½ whorls, with roundish malleated pits throughout. Teleoconch with strong, regularly spaced radial ribs; earlier whorls ribs regularly strong, last three whorls’ ribs slightly elevated, weakly sinuous at angulation, and then thinner and lower near suture. Ribs with 6–7 ribs/0.5 mm on penultimate whorl; 10–11 ribs/0.5 mm on last whorl towards aperture. Spiral striations very thin and fine, appearing from teleoconch to aperture. Last whorl narrower than penultimate whorl and displaced rightward relative to peristome position. Constriction inconspicuous externally and internally. Aperture circular bearing, with three barriers: parietalis short, very weak, low knob-like; palatalis elongate horizontally and form tall ridge; columellaris prominent, continuous with peristome, slightly deflected downward toward palatal wall (Fig. 11D). Peristome broadly expanded, double; outer peristome broader than inner peristome. Lip thickened, multi-layered; parietal callus thickened and expanded. Columella straight, thickened. Umbilicus closed.

###### Distribution.

*Pagodapalaina suratensis* occurs in several areas of southern Thailand (Panha and Burch 2005). It has recently been recorded from limestone outcrops in the Tanintharyi Region, southern Myanmar (Fig. 1A).

###### Remarks.

This is the first record of this species in Myanmar. *Diplommatina somsakpanhai* sp. nov. was also found syntopic with this species. The new species differs in having opposite shell coiling, an ovate shell shape, and a very large, thickened shell aperture.

### Synoptic list of *Diplommatina* species recorded from Myanmar

This synoptic list includes all the *Diplommatina* species that have their type locality within the geographic area of present-day Myanmar [formerly Burma], and the species subsequently or provisionally reported from Myanmar. All the species are listed alphabetically where their original publication was provided. In all instances, the original literature was confirmed for authorship and date, page numbers of the original description and illustrations, and type locality to ensure the accuracy of the entries. The usage of the name, necessary references that provided descriptions or images of the species, and taxonomic treatment articles that mentioned the species names are also listed. The depository information of the name-bearing types (holotype, lectotype, or syntype(s)) is provided and is illustrated when possible. However, in the cases where the name-bearing types could not be traced, topotypic or authentic reference specimens are illustrated instead for further comparison and identification. In some instances, information about the authorship, type series, and type locality is discussed in the remarks section. If necessary, remarks are given on the status of type specimens, authorships, availability of name, new replacement name, notes on the type locality, and other valuable comments.

Nine nominal species described from either ‘Munipur’ or the ‘Naga Hills’ were provisionally listed as occurring in ‘Burma’ by Gude (1921): *D. ambigua* Godwin-Austen, 1892, *D. animula* Godwin-Austen, 1892, *D. butleri* Godwin-Austen, 1892, *D. commutata* Godwin-Austen, 1892, *D. compacta* Godwin-Austen, 1892, *D. lapillus* Godwin-Austen, 1892, *D. munipurensis* Godwin-Austen, 1892, *D. thomsoni* Godwin-Austen, 1892, and *D. tumida laisenensis* Gude, 1921. Although ‘Munipur’ was formerly part of the Burmese Empire, it came under British control following the First Anglo-Burmese War (1824–1826) and is now a state in northeastern India. The ‘Naga Hills’ are primarily located in northeastern India, including the states of Assam, Arunachal Pradesh, and Nagaland, although the range extends into northern Myanmar, likely within the Naga Self-Administered Zone and Sagaing Region (Raheem et al. 2014: 270). At present, there is no definitive evidence confirming the occurrence of these nine species in present-day Myanmar. Nevertheless, they are included here following Gude (1921), pending future intensive field surveys that may clarify their distributional ranges within Myanmar. Their inclusion is further justified because future fieldwork in northern Myanmar may yet confirm their presence, and documenting these taxa now ensures that relevant information will be readily available when these species are rediscovered. In addition, several of these species have never been illustrated, and the images of the type specimens presented here provide valuable reference that may assist future systematic studies, particularly for researchers working in northeastern India and northern Myanmar.

#### 9 Diplommatina affinis

Taxon classificationAnimaliaArchitaenioglossaDiplommatinidae

Theobald, 1870

13EA51FC-53BF-51B4-90D9-365D553A5680

Fig. 15A

Diplommatina affinis Theobald, 1870: 398. Type locality: Shan States. Pfeiffer 1875: 70. Theobald 1876: 41. Godwin-Austen 1884: pl. 49, fig. 3. Godwin-Austen 1886: 181, 182. Gude 1921: 302.
Diplommatina (Eudiplommatina) affinis —Kobelt and Möllendorff 1898: 135.
Diplommatina (Diplommatina) affinis —Kobelt 1902: 424.

##### Type material examined.

Unknown or probably lost.

**Figure 15.**
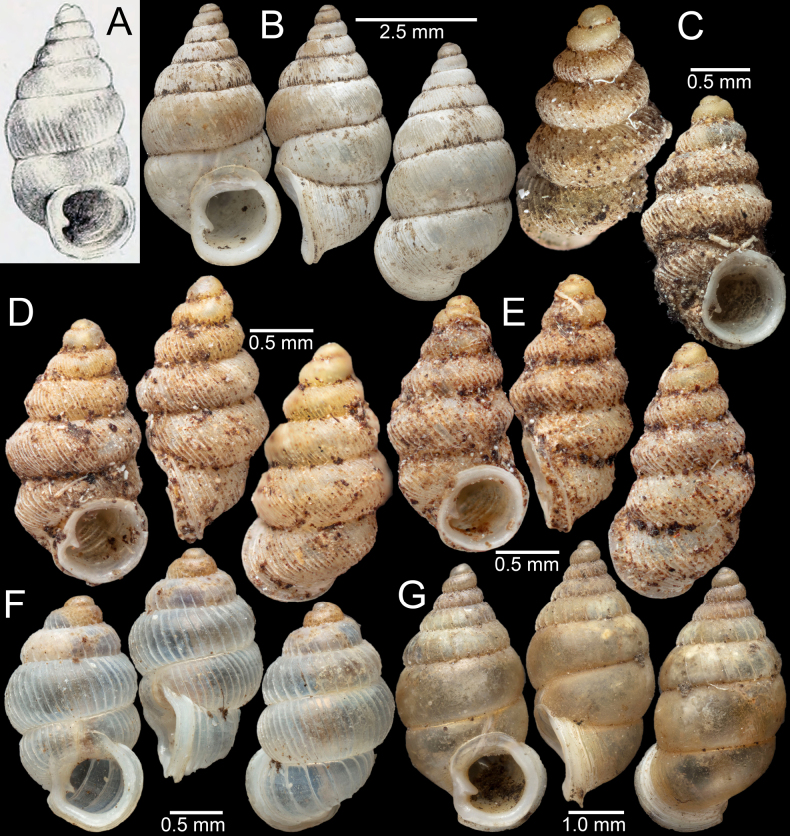
**A**. *Diplommatina affinis* Theobald, 1870 (after Godwin-Austen 1884: pl. 49, fig. 3); **B**. *Diplommatina ambigua* Godwin-Austen, 1892, syntype NHMUK 1903.7.1.2124 from South of Burrail Range; **C–E**. *Diplommatina angulata* Theobald & Stoliczka, 1872: **C, D**. Syntypes NHMUK 1888.12.4.232–241 from Chouktalon, Moulmein; **E**. Specimen NHMUK 20240015 from Moulmein, Burma; **F**. *Diplommatina animula* Godwin-Austin, 1892, syntype NHMUK 1903.7.1.2164 from Prowi, Lahupa, Naga Hills; **G**. *Diplommatina butleri* Godwin-Austin, 1892, syntype NHMUK 1903.7.1.2128 from Laisen Peak (Trig. Station), Munipur.

##### Diagnosis.

Shell dextral, ovate-turreted. Whorls 7, increasing regularly; ribs weak, slightly prominent on last whorl. Aperture ovate; columellar straight, columellaris small. Peristome double; outer peristome slightly expanded.

##### Distribution.

The species was known from Shan State (Theobald 1870) and was subsequently recorded from Modi Taung in the Arakan Hills, Rakhine State, Myanmar, by Godwin-Austen (1886).

##### Remarks.

This is one of four diplommatinid species described by Theobald (1870) for which the type series could not be located in either the NHM, London, nor the NMW, Cardiff (T. White, B. Rowson, and J. Gallichan, pers. comm.). As the type specimens could not be examined, the diagnosis provided here is based on the original description and the illustration by Godwin-Austen (1884: pl. 49, fig. 3).

#### 10 Diplommatina ambigua

Taxon classificationAnimaliaArchitaenioglossaDiplommatinidae

Godwin-Austen, 1892

08F56A45-71E3-525C-A378-4A852C0AE2F1

Fig. 15B

Diplommatina ambigua Godwin-Austen, 1892: 513. Type locality: South of Burrail Range, Munipur. Godwin-Austen 1897: 8, pl. 64, fig. 2. Gude 1921: 302.
Diplommatina (Eudiplommatina) ambigua —Kobelt and Möllendorff 1898: 135.
Diplommatina (Diplommatina) ambigua —Kobelt 1902: 424.

##### Type material examined.

India • ***Syntypes***. NHMUK 1903.7.1.2124, (7 shells; Fig. 15B) from South of Burrail Range (figured in Godwin-Austen 1897: pl. 64, fig. 2); Godwin-Austen coll.

##### Diagnosis.

Shell dextral, ovate fusiform, dull, whitish. Whorls ~7–8, well rounded; spire elevated conical; suture wide, shallow. Teleoconch: earlier whorls with weak, narrowly and equally spaced radial ribs; penultimate and last whorls with slightly wide-spaced ribs. Penultimate whorl convex, equal in width to last whorl. Aperture circular; columellaris strong, directed downward. Peristome expanded, double, multi-layered; inner peristome slightly broader than outer peristome; parietal callus expanded. Columella straight; umbilicus closed.

##### Distribution.

This species was previously known only from its type locality in the Burrail Range, Manipur State, India. The record from Myanmar lacks a precise locality (Gude 1921), and the presence there has not yet been reconfirmed.

#### 11 Diplommatina angulata

Taxon classificationAnimaliaArchitaenioglossaDiplommatinidae

Theobald & Stoliczka, 1872

502D1E16-EBB8-55D9-906A-1BAC163D796D

Fig. 15C–E

Diplommatina angulata Theobald & Stoliczka, 1872: 331, pl. 11, fig. 3. Type locality: Prope Moulmain, provincia Martaban. Hanley and Theobald 1875: 55, pl. 140, fig. 7. Pfeiffer 1875: 78. Nevill 1878: 285. Godwin-Austen 1884: pl. 49, figs 5, 5a. Godwin-Austen 1886: 184. Gude 1921: 302, 303.
Palaiana angulata —Theobald 1876: 43.
Diplommatina (Eudiplommatina) angulata —Kobelt and Möllendorff 1898: 135.
Diplommatina (Diplommatina) angulata —Kobelt 1902: 424, 425.

##### Type material examined.

Myanmar • ***Syntypes***. NHMUK 1888.12.4.232–241 (10 shells; Fig. 15C, D) from Chouktalon, Moulmein; Theobald coll.

##### Other material.

Myanmar • NHMUK 20240015 (3 shells; Fig. 15E) from Moulmein, Burma; Sykes coll. • NHMUK 20240016 (1 shell) from Burma; Moulmein; Salisbury coll. • NHMUK 20240017 (4 shells) from India; MacAndrew coll.

##### Diagnosis.

Shell dextral, conical-fusiform, translucent, whitish to yellowish. Whorls ~5–6, angular; spire conical; suture wide, deep. Teleoconch with wide, equally spaced radial ribs throughout. Penultimate whorl well rounded, wider than last whorl. Aperture rounded; columellaris strong, distinct, directed downward. Peristome expanded, double, multi-layered. Umbilicus narrowly open.

##### Distribution.

The species is currently known from its type locality, corresponding to present-day Mawlamyine District in Mon State, Myanmar.

##### Remarks.

*Diplommatina angulata* Stanisic, 2010, described from Queensland, Australia, is a junior primary homonym of *D. angulata* Theobald & Stoliczka, 1872, from Myanmar (Theobald and Stoliczka 1872; Stanisic et al. 2010; MolluscaBase 2025). The two nominal taxa are clearly distinct in shell morphology and distributional range (Stanisic et al. 2010: 74, 75 and text figure). The younger name is invalid under the ICZN (1999: Arts 57.2, 60.3). As no available junior synonym exists, a replacement name is required to maintain nomenclatural stability. We therefore propose *Diplommatina stanisici* Tongkerd, nom. nov., as the new substitute name for the Australian species. The holotype of *D. stanisici* nom. nov. is the holotype of the replaced name (QM MO50138), and is deposited in the Queensland Museum, Australia.

#### 12 Diplommatina animula

Taxon classificationAnimaliaArchitaenioglossaDiplommatinidae

Godwin-Austen, 1892

5EE88336-451E-5472-A7A7-F72128CABD30

Fig. 15F

Diplommatina animula Godwin-Austin, 1892: 516, 517. Type locality: Prowi, Lahupa Naga Hills, Munipur. Godwin-Austin 1897: 12, 13 pl. 66, fig. 2. Gude 1921: 345.
Diplommatina (Sinica) animula —Kobelt and Möllendorff 1898: 139. Kobelt 1902: 455.

##### Type material examined.

India • ***Syntype***. NHMUK 1903.7.1.2164 (1 shell; Fig. 15F) from Prowi, Lahupa, Naga Hills (figured in Godwin-Austen 1897: pl. 66, fig. 2); Godwin-Austen coll.

##### Diagnosis.

Shell dextral, ovate-fusiform, translucent, whitish. Whorls ~5, rounded; spire depressed conical; suture wide, deep. Teleoconch with densely placed, equally spaced, strong radial ribs throughout. Last three whorls nearly equal in width; penultimate whorl slightly wider than others. Aperture rounded; columellaris weak, indistinct. Peristome expanded, double, multi-layered; outer peristome expanded beyond inner peristome. Columella straight, with sinulus below. Umbilicus narrowly open.

##### Distribution.

This species was previously known only from its type locality in Prowi, Lahupa-Naga Hills, Manipur State, India. The record from Myanmar does not provide a precise locality (Gude 1921).

#### 13 Diplommatina butleri

Taxon classificationAnimaliaArchitaenioglossaDiplommatinidae

Godwin-Austen, 1892

E20579E0-8386-57CA-B75E-5919B7781B2F

Fig. 15G

Diplommatina butleri Godwin-Austin, 1892: 512, 513. Type locality: Laisen Peak, Munipur. Godwin-Austin 1897: 6, 7, pl. 64, fig. 9. Gude 1921: 305, 306. McGhie 2008: 9.
Diplommatina (Eudiplommatina) butleri —Kobelt and Möllendorff 1898: 135.
Diplommatina (Diplommatina) butleri —Kobelt 1902: 427.

##### Type material examined.

India • ***Syntypes***. NHMUK 1903.7.1.2128 (6 shells; Fig. 15G) from Laisen Peak (Trig. Station), Munipur (figured in Godwin-Austen 1897: pl. 64, fig. 9); Godwin-Austen coll.

##### Diagnosis.

Shell dextral, ovate-fusiform, translucent, pale brownish. Whorls ~7–8, well rounded; spire elevated conical; suture wide, shallow. Teleoconch: earlier whorls with weak, widely, and equally spaced radial ribs; penultimate whorl and last whorl generally smooth. Penultimate whorl convex, equal in width to last whorl. Aperture circular; columellaris very strong, directed downward. Peristome expanded, double, multi-layered; outer peristome slightly broader than inner peristome. Columella angulate, with distinct sinulus below. Umbilicus closed.

##### Distribution.

This species was originally known from its type locality at Laiven Peak, Manipur State, India. It has also been recorded from several other localities, including the Lahupa-Naga Hills, Kezakenomih, and Klang Sing in the Naga Hills, India (Godwin-Austen 1892; Gude 1921). The record from Myanmar, however, lacks a precise locality and is cited only as ‘Kyengdwen, Burmah’, which likely refers to the Chindwin River valley in Sagaing Region, Myanmar.

##### Remarks.

Three syntypes are also housed in the Manchester Museum, The University of Manchester (EE.3511/109) (McGhie 2008). This species closely resembles *D. ambigua* in shell form but differs in having widely and regularly spaced radial ribs on the earlier whorls, and the penultimate and last whorls are generally smooth. In contrast, *D. ambigua* (Fig. 15B) possesses narrowly and regularly spaced radial ribs on the earlier whorls, and the penultimate and last whorls bear widely spaced ribs.

#### 14 Diplommatina commutata

Taxon classificationAnimaliaArchitaenioglossaDiplommatinidae

Godwin-Austen, 1892

E8ED2E64-FA4C-5446-99CE-A6850A5889AF

Fig. 16B

Diplommatina commutata Godwin-Austen, 1892: 513. Type locality: Prowi, Lahupa Naga Hills. Godwin-Austen 1897: 8, pl. 64, fig. 4. Gude 1921: 306, 307.
Diplommatina (Eudiplommatina) commutata —Kobelt and Möllendorff 1898: 136.
Diplommatina (Diplommatina) commutata —Kobelt 1902: 428.

##### Type material examined.

**Figure 16.**
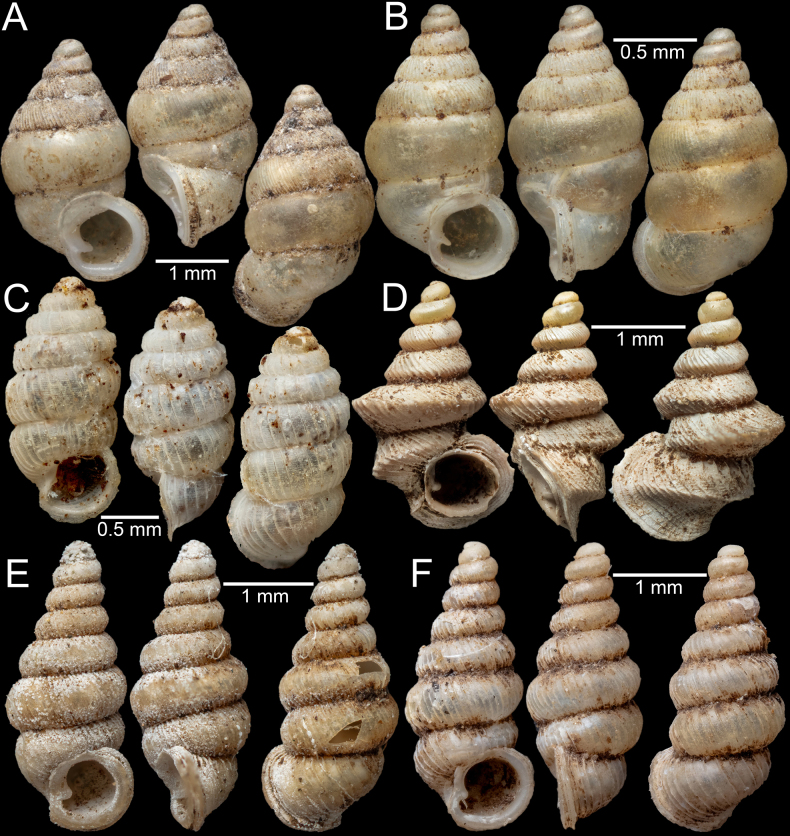
**A**. *Diplommatina compacta* Godwin-Austen, 1892, syntype NHMUK 1903.7.1.2179 from South of Barak, Munipur; **B**. *Diplommatina commutata* Godwin-Austen, 1892, syntype NHMUK 1903.7.1.2126 from Prowi, Lahupa, Naga Hills; **C**. *Diplommatina edentata* Godwin-Austen, 1886, syntype NHMUK 1903.7.1.2201 from Moulmein, Tenasserim; **D**. *Diplommatina crispata* Stoliczka, 1871, syntypes NHMUK 1903.7.1.2200 from Moulmein, Tenasserim; **E, F**. *Diplommatina exilis* Blanford, 1863: **E**. Syntype NHMUK 1903.7.1.2207 from Mya Leit Doung, Ava; **F**. Syntypes NHMUK 1906.4.4.16 from Mya Leit Doung, Ava.

India • ***Syntypes***. NHMUK 1903.7.1.2126 (7 shells; Fig. 16B) from Prowi, Lahupa, Naga Hills (figured in Godwin-Austen 1897: pl. 64, fig. 4); Godwin-Austen coll.

##### Diagnosis.

Shell dextral, ovate-fusiform, translucent, pale brownish. Whorls ~7, well rounded; spire elevated-conical; suture wide, shallow. Teleoconch: earlier whorls with weak, narrowly and equally spaced radial ribs; penultimate and last whorls with very weak radial ribs to nearly smooth surface. Penultimate whorl convex, slightly wider than last whorl. Aperture circular; columellaris very strong, sharp, directed downward. Peristome expanded, double, multi-layered; outer peristome slightly broader than inner peristome. Columella curved; umbilicus closed.

##### Distribution.

This species was originally known from its type locality at Prowi, Lahupa-Naga Hills, Manipur State, India, and has also been recorded from Tellizo Peak, Anghami–Naga Hills, Manipur State, India. The record from Myanmar, however, lacks an exact locality (Gude 1921), and the presence there has not yet been reconfirmed.

#### 15 Diplommatina compacta

Taxon classificationAnimaliaArchitaenioglossaDiplommatinidae

Godwin-Austen, 1892

DA36122F-AD24-5A8D-85E2-DD61A256335B

Fig. 16A

Diplommatina compacta Godwin-Austen, 1892: 515, 516. Type locality: South of Barak in Munipur. Godwin-Austen 1897: 11, 12, pl. 65, figs 7, 7a, 7b. Gude 1921: 340. McGhie 2008: 11.
Diplommatina (Metadiancta) compacta —Kobelt and Möllendorff 1898: 138. Kobelt 1902: 449.

##### Type material examined.

India • ***Syntypes***. NHMUK 1903.7.1.2179 (28 shells; Fig. 16A) from South of Barak, Munipur (figured in Godwin-Austen 1897: pl. 65, figs 7, 7a); Godwin-Austen coll.

##### Diagnosis.

Shell dextral, ovate-fusiform, translucent, whitish. Whorls ~7, well rounded; spire elevated-conical; suture wide, shallow. Teleoconch: earlier whorls with weak, narrowly and equally spaced radial ribs; penultimate and last whorls with very weak radial ribs to nearly smooth surface. Penultimate whorl convex, wider than last whorl. Aperture circular; columellaris very strong, directed downward. Peristome expanded, double, multi-layered; outer peristome slightly broader than inner peristome; parietal callus thickened, expanded. Columella curved; umbilicus closed.

##### Distribution.

This species is known only from its type locality in Barak, Manipur State, India. The record from Myanmar, however, lacks a precise locality (Gude 1921), and the presence there has not yet been reconfirmed.

##### Remarks.

There are four additional syntype shells housed in The Manchester Museum, University of Manchester (EE.3511/99) (McGhie 2008).

#### 16 Diplommatina crispata

Taxon classificationAnimaliaArchitaenioglossaDiplommatinidae

Stoliczka, 1871

819FF825-94A3-508B-B1B7-7EDEB2E86905

Fig. 16D

Diplommatina (Palaina) crispata Stoliczka, 1871: 153, pl. 6, fig. 4. Type locality: Damotha, prope Moulmein. Pfeiffer 1875: 91, 92. Nevill 1878: 289. Gude 1921: 308, 309.
Palaina crispata —Theobald 1876: 43.
Diplommatina crispata —Hanley and Theobald 1876: 56, pl. 141, fig. 6. Godwin-Austen 1884: pl. 49, figs 4, 4a, 4b. Godwin-Austen 1886: 183.
Diplommatina (Eudiplommatina) crispata —Kobelt and Möllendorff 1898: 136.
Diplommatina (Diplommatina) crispata —Kobelt 1902: 429.

##### Type material examined.

Myanmar • ***Syntypes***. NHMUK 1903.7.1.2200 (2 shells; Fig. 16D) from Moulmein, Tenasserim (figured in Godwin-Austen 1884: pl. 49, figs 4, 4a); Godwin-Austen coll.

##### Diagnosis.

Shell dextral, elongate-fusiform, dull, and pale brownish to pale yellowish. Whorls ~6–7; earlier whorls rounded-angular then becoming stronger on last two whorls; spire elongate-conical; suture wide, deep. Teleoconch with equally spaced radial ribs throughout: earlier whorls regular, strong radial ribs; last two whorls with strong, tall, sinuous radial ribs at angulation. Spiral striations fine, conspicuously present on last two whorls. Penultimate whorl much wider than last whorl; last whorl much displaced to right relative to peristome position. Aperture rounded; columellaris distinct, directed downward. Peristome expanded, double; outer peristome more broadly expanded than inner peristome, multi-layered; inner peristome slightly expanded, elevated. Umbilicus very narrow.

##### Distribution.

The species is currently known only from its type locality, ‘Damotha, near Moulmein’, which corresponds to the modern Damathat Cave (16°30'21.8"N, 97°49'11.5"E) in Mawlamyine, Mon State, Myanmar.

##### Remarks.

*Diplommatina angulifera* Bavay & Dautzenberg, 1912 from Vietnam and *D. angulifera umpangensis* Panha, 1998 from Thailand exhibit shell shapes and sculptures similar to those of *D. crispata*, and they are likely conspecific. Additionally, *D. crispata khaochamaoensis* Panha, Kanchanasaka & Burch, 2002 from Chanthaburi Province, Thailand which is located approximately 600 km southeast of the type locality, differs slightly from the nominotypical subspecies. It possesses a less prominent angulation on the last whorl, whereas the nominotypical subspecies exhibits a more pronounced and sharply defined peripheral keel on the penultimate whorl that expands to nearly the same extent as that of the last whorl.

#### 17 Diplommatina edentula

Taxon classificationAnimaliaArchitaenioglossaDiplommatinidae

Godwin-Austen, 1886

54AC760F-3564-5A7F-B088-9C61419ADE48

Fig. 16C

Diplommatina edentula Godwin-Austen, 1884: pl. 49, figs 7, 7a. [nomen nudum]
Diplommatina edentula Godwin-Austen, 1886: 185. Type locality: Moulmain [Mawlamyine, Myanmar]. Gude 1921: 312.
Diplommatina (Eudiplommatina) edentula —Kobelt and Möllendorff 1898: 136.
Diplommatina (Diplommatina) edentula —Kobelt 1902: 431.

##### Type material examined.

Myanmar • ***Syntypes***. NHMUK 1903.7.1.2201 (2 shells; Fig. 16C) from Moulmein, Tenasserim (figured in Godwin-Austen 1884: pl. 49, figs 7, 7a); Godwin-Austen coll.

##### Diagnosis.

Shell tiny, dextral, pupate-fusiform, translucent, pale yellowish to whitish. Whorls ~6, slightly shouldered to rounded; spire ovate-conical; suture deep. Teleoconch with equally spaced, strong radial ribs throughout. Spiral striations thin and appearing throughout. Last three whorls nearly equal in width; penultimate whorl slightly less inflated than others. Aperture ovate; columellaris weak, not prominent. Peristome expanded; lip slightly thickened, multi-layered. Umbilicus closed.

##### Distribution.

This species was previously known only from its type locality in Mawlamyine, Mon State, Myanmar (Godwin-Austen 1886; Gude 1921).

##### Remarks.

The name *edentula* was first introduced by Godwin-Austen (1884) in ‘Land and Freshwater Mollusca of India’; however, it was published without a description, definition, or indication, as only shell illustrations and the collection locality were provided. Accordingly, the name does not meet the criteria of availability under the ICZN (1999: Art. 12.2) and was therefore unavailable in Godwin-Austen (1884). Subsequently, in the same series, Godwin-Austen (1886) published a full description of the taxon under the same name, thereby rendering *edentula* an available name from that publication.

#### 18 Diplommatina exilis

Taxon classificationAnimaliaArchitaenioglossaDiplommatinidae

Blanford, 1863

ADDB7F95-B9B9-5F4F-B44D-FFEB31C1059D

**Figure 17.**
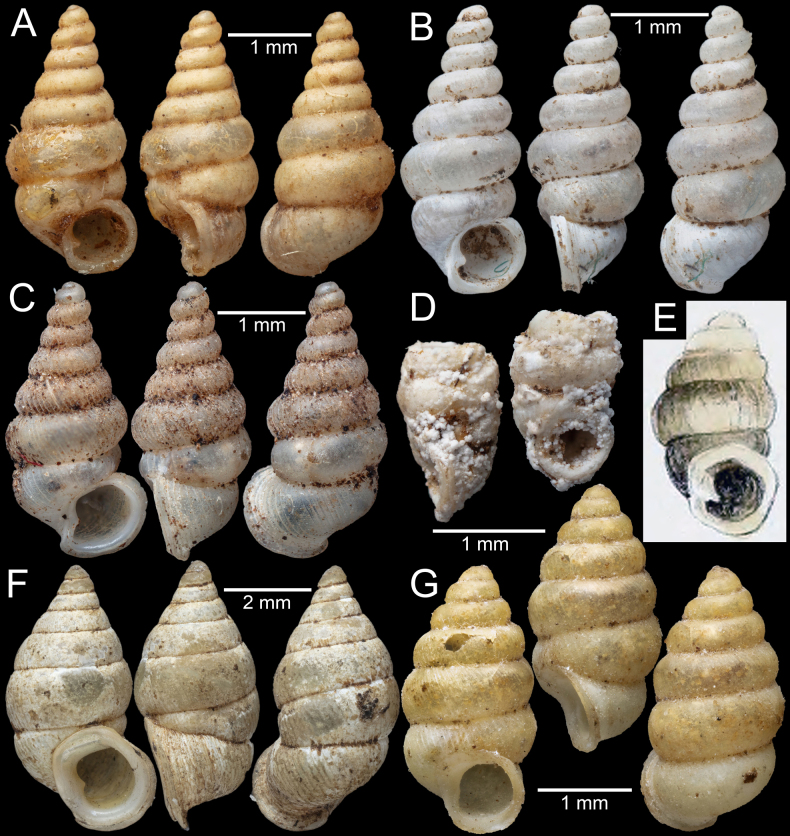
**A, B**. *Diplommatina exilis* Blanford, 1863: **A**. Specimen NHMUK 1888.12.4.163–166 from Myaleit Doung, Ava; **B**. Specimen NHMUK 1903.7.1.2277 from Ava, Burma; **C**. *Diplommatina exserta* Godwin-Austen, 1886, syntypes NHMUK 1903.7.1.2208 from Damathat, Moulmein; **D, E**. *Diplommatina henzadaensis* Godwin-Austen, 1886: **D**. Holotype NHMUK 1906.4.4.22 from Khyoung Gyoung nulla, Henzada; **E**. After Godwin-Austen (1884: pl. 46, fig. 6); **F**. *Diplommatina lapillus* Godwin-Austen, 1892, syntype NHMUK 1903.7.1.2151 from no locality data; **G**. *Diplommatina munipurensis* Godwin-Austen, 1892, syntype NHMUK 1903.7.1.2165 from South Barak, Munipur.

Figs 16E, 16F, 17A, 17B

Diplommatina exilis Blanford, 1863: 325. Type locality: Mya Leit Douang, Ava. Pfeiffer 1865: 10. Godwin-Austen 1868: 84, pl. 4, figs 3, 3a. Theobald and Stoliczka 1872: 331. Hanley and Theobald 1875: 49, pl. 119, fig. 10. Pfeiffer 1875: 77. Theobald 1876: 42. Nevill 1878: 284. Godwin-Austen 1884: pl. 49, fig. 1. Godwin-Austen 1886: 180. Gude 1921: 313.
Diplommatina (Eudiplommatina) exilis —Kobelt and Möllendorff 1898: 136.
Diplommatina (Diplommatina) exilis —Kobelt 1902: 432.

##### Type material examined.

Myanmar • ***Syntypes***. NHMUK 1906.4.4.16 (6 shells; Fig. 16F) from Mya Leit Doung, Ava; W.T. Blanford coll. • ***Syntype***. NHMUK 1903.7.1.2207 (1 shell; Fig. 16E) from Mya Leit Doung, Ava (figured in Godwin-Austen 1884: pl. 49, fig. 1); Godwin-Austen coll.

##### Other material.

Myanmar • NHMUK 1888.12.4.163–166 (4 shells; Fig. 17A) from Myaleit Doung, Ava; Theobald coll. • NHMUK 1903.7.1.2277 (1 shell; Fig. 17B) from Ava, Burma; Godwin-Austen coll. • NHMUK 20240018 (2 shells) from Ava; Sykes coll. • NHMUK 20240019 (4 shells) from India (?); Cuming coll.

##### Diagnosis.

Shell dextral, elongate-conical fusiform, dull to translucent, whitish to yellowish. Whorls ~7–8, well rounded; spire elongate-conical; suture wide, deep. Teleoconch with equally spaced, weak to strong radial ribs throughout. Penultimate whorl wider than preceding and last whorls. Aperture rounded; columellaris strong, distinct, directed downward. Peristome expanded, double; lip thickened, multi-layered. Umbilicus closed.

##### Distribution.

The species is currently known only from its type locality, ‘Mya Leit Douang, Ava’, which likely refers to the Myaleit Mountains in the Mandalay Region, Myanmar.

#### 19 Diplommatina exserta

Taxon classificationAnimaliaArchitaenioglossaDiplommatinidae

Godwin-Austen, 1886

123EA453-004A-5BCE-B5BC-9D74CEEEE193

Fig. 17C

Diplommatina exilis var. exserta (nom. nud.) Nevill, 1878: 284. [nomen nudum]
Diplommatina exserta Godwin-Austen, 1884: pl. 49, figs 2, 2a. [nomen nudum]
Diplommatina exserta Godwin-Austen, 1886: 184, 185. Type locality: Damotha Cave, Moulmein. Gude 1921: 314. Budha et al. 2017: 8, figs 3b, 9b.
Diplommatina (Eudiplommatina) exserta —Kobelt and Möllendorff 1898: 136.
Diplommatina (Diplommatina) exserta —Kobelt 1902: 432. Budha et al. 2015: 6.

##### Type material examined.

Myanmar • ***Syntypes***. NHMUK 1903.7.1.2208 (4 shells; Fig. 17C) from Damathat, Moulmein (figured in Godwin-Austen 1884: pl. 49, figs 2, 2a); Godwin-Austen coll.

##### Diagnosis.

Shell dextral, turreted-fusiform, translucent, whitish. Whorls ~7–8, well rounded; spire elongate-conical; suture wide, deep. Teleoconch with slightly strong, densely and equally spaced radial ribs throughout. Spiral striations very thin and appearing throughout. Penultimate whorl slightly wider than preceding and last whorls. Aperture rounded, little tilted from columellar axis; columellaris very weak. Peristome expanded, double; basal edge slightly sinuous, protruding; outer peristome slightly broader than inner peristome, multi-layered. Umbilicus closed.

##### Distribution.

The species is currently known from its type locality in Mon State and was subsequently recorded from Nepal, approximately 1900 km from the type locality (Gude 1921; Budha et al. 2015, 2017).

##### Remarks.

This taxon name was first mentioned by Godwin-Austen (1884) solely in a figure caption, accompanied only by the collection locality. This treatment lacked a description, definition, and indication, and thus did not meet the requirements for availability under the ICZN (1999: Art. 12). Godwin-Austen (1886) subsequently published a full description of the taxon, where the name exserta became available.

The specimens from Nepal differ slightly from the syntype, having a somewhat narrower penultimate whorl at the lip, a strong columellar tooth, and a more inflated penultimate whorl compared to the preceding whorl. In contrast, the syntype shows a very weak columellar tooth, a narrower penultimate whorl at the lip, and a penultimate whorl nearly equal in swelling to the preceding whorl. These differences suggest that the specimens from Nepal may belong to a distinct species.

#### 20 Diplommatina henzadaensis

Taxon classificationAnimaliaArchitaenioglossaDiplommatinidae

Godwin-Austen, 1886

0D257729-8F27-5BCF-90A3-AFF42FCBA9B0

Fig. 17D, E

Diplommatina henzadaensis Godwin-Austen, 1884: pl. 46, figs 6, 6a. [nomen nudum]
Diplommatina henzadaensis Godwin-Austen, 1886: 179, 180. Type locality: Kyoung Gyoung Nulla, Henzada, Pegu. Gude 1921: 318.
Diplommatina (Eudiplommatina) henzadaensis —Kobelt and Möllendorff 1898: 137.
Diplommatina (Diplommatina) henzadaensis —Kobelt 1902: 434, 435.

##### Type material examined.

Myanmar • ***Holotype***. NHMUK 1906.4.4.22 (Fig. 17D) from Khyoung Gyoung-nulla, Henzada; W.T. Blanford coll.

##### Diagnosis.

Shell dextral, ovate-turreted. Six whorls, rounded; antepenultimate whorl largest; sculptured with fine, widely spaced radial ribs. Spire convex; apex blunt; suture well marked. Aperture circular. Peristome double, continuous, thickened; columellaris well-developed.

##### Distribution.

This species is currently known only from its type locality, ‘Kyoung Gyoung Nulla, Henzada, Pegu’, which likely corresponds to the modern Kyaunggon Township, Pathein District, Ayeyarwady Region, Myanmar.

##### Remarks.

This taxon was first mentioned by Godwin-Austen (1884) without a formal description, definition, or indication; only shell illustrations and the collection locality were provided. Consequently, the name does not meet the criteria for availability under the ICZN (1999: Art. 12.2) and is therefore unavailable. Godwin-Austen (1886) subsequently published a complete description of the taxon under the same name, thereby making ‘*henzadaensis*’ available. In the original description, Godwin-Austen (1886) clearly stated that only a single specimen existed in the Blanford collection. The holotype, which is affected by Byne’s disease and consequently fragile, is illustrated here for the first time.

#### 21 Diplommatina lapillus

Taxon classificationAnimaliaArchitaenioglossaDiplommatinidae

Godwin-Austen, 1892

CD057A9E-AF51-5C69-AE8A-E66A78D260B5

Fig. 17F

Diplommatina lapillus Godwin-Austen, 1892: 515. Type locality: Kopamedza Peak, Lahupa Naga Hills, 8375 ft. Godwin-Austen 1897: 11, pl. 65, figs 6, 6a. Gude 1921: 342.
Diplommatina (Metadiancta) lapillus —Kobelt and Möllendorff 1898: 139. Kobelt 1902: 450.

##### Type material examined.

India • ***Syntypes***. NHMUK 1903.7.1.2151 (2 shells; Fig. 17F) from Kopamedza Peak, Naga Hills (figured in Godwin-Austen 1897: pl. 65, figs 6, 6a); Godwin-Austen coll.

##### Diagnosis.

Shell dextral, ovate-fusiform, dull, whitish. Whorls ~7, well rounded; spire elevated-conical; suture wide, shallow. Teleoconch with very weak, narrowly and equally spaced radial ribs throughout. Penultimate whorl convex, equal in width to last whorl. Aperture circular; columellaris weak, small, directed downward. Peristome broadly expanded, double, multi-layered; outer peristome slightly broader than inner peristome; parietal callus thickened, expanded, nearly attached to suture. Columella straight; umbilicus closed.

##### Distribution.

This species is known from its type locality at Kopamedza Peak, Lahupa–Naga Hills, Manipur State, India, as well as from locations north of the Barail Range within the same state. Records from Myanmar lack precise locality data (Gude 1921), and the presence there has not yet been reconfirmed.

#### 22 Diplommatina munipurensis

Taxon classificationAnimaliaArchitaenioglossaDiplommatinidae

Godwin-Austen, 1892

A8BCFBF9-A50B-5AF0-9126-31B8A62F6CCB

Fig. 17G

Diplommatina munipurensis Godwin-Austen, 1892: 518. Type locality: South of Barak River, between the Mao villages and Munipur. Godwin-Austen 1897: 14, pl. 66, fig. 6. Gude 1921: 323, 324. Budha et al. 2017: 13, 15, figs 5b, 9h. McGhie 2008: 24.
Diplommatina (Eudiplommatina) munipurensis —Kobelt and Möllendorff 1898: 137.
Diplommatina (Diplommatina) munipurensis —Kobelt 1902: 438. Budha et al. 2015: 7.

##### Type material examined.

India • ***Syntypes***. NHMUK 1903.7.1.2165 (10 shells; Fig. 17G) from South Barak, Munipur (figured in Godwin-Austen 1897: pl. 66, fig. 6); Godwin-Austen coll.

##### Diagnosis.

Shell dextral, elongate, pupate-fusiform, translucent, pale yellowish. Whorls ~6–7, rounded; spire elongate-conical; suture wide, deep. Teleoconch with weak, irregularly spaced radial ribs throughout. Penultimate whorl equal to or slightly wider than last whorl. Aperture ovate; columellaris very weak, indistinct. Peristome expanded, multi-layered; lip slightly thickened; parietal callus thin, expanded. Columella thickened, straight; umbilicus closed.

##### Distribution.

This species was originally known only from its type locality in Manipur State, India. It was later reported from several localities in Bagmati and Sudurpashchim provinces, Nepal (Budha et al. 2015, 2017). Records from Myanmar, however, lack precise locality data (Gude 1921), and the presence there has not yet been reconfirmed.

##### Remarks.

There are two syntypes housed in The Manchester Museum, University of Manchester (EE.3511/87) (McGhie 2008).

#### 23 Diplommatina nana

Taxon classificationAnimaliaArchitaenioglossaDiplommatinidae

Blanford, 1865

8EC55CF3-1F60-5D49-8D87-0942A97B47DB

Fig. 18A–E

Diplommatina nana Blanford, 1865: 85. Type locality: Akoutoung, Thondoung and Yenandoung in Henzada district, Pegu. Godwin-Austen 1868: 84, pl. 4, figs 4, 4a. Theobald and Stoliczka 1872: 331. Hanley and Theobald 1875: 55, pl. 140, fig. 1. Pfeiffer 1875: 75. Theobald 1876: 42. Nevill, 1878: 285. Godwin-Austen 1884: pl. 49, figs 6, 6a. Godwin-Austen 1886: 181. Gude 1921: 324.
Diplommatina (Eudiplommatina) nana —Kobelt and Möllendorff 1898: 137.
Diplommatina (Diplommatina) nana —Kobelt 1902: 439.

##### Type material examined.

**Figure 18.**
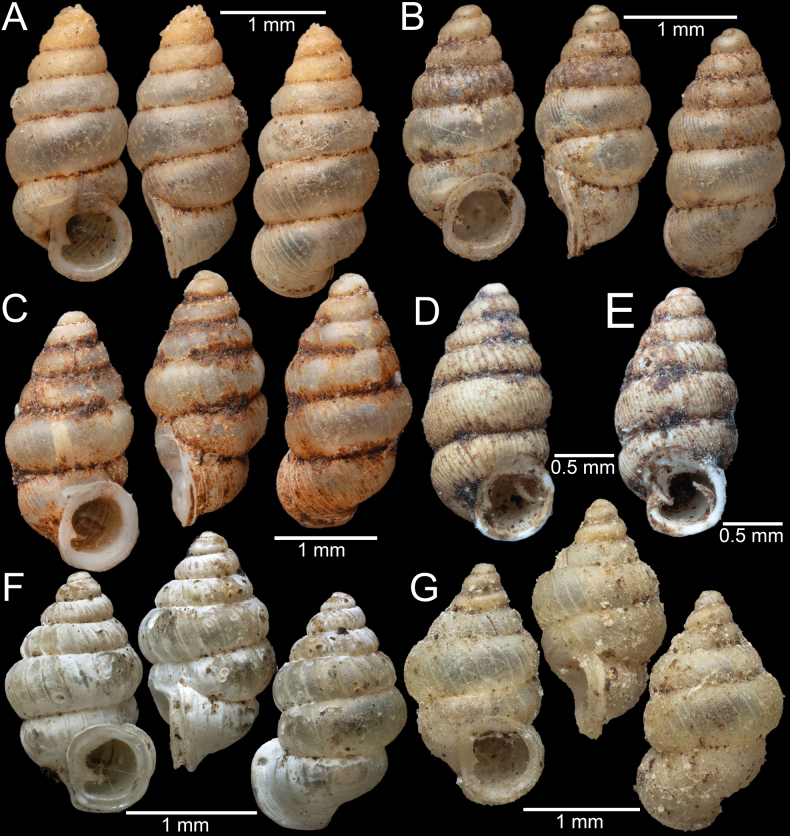
**A–E**. *Diplommatina nana* Blanford, 1865: **A**. Specimen NHMUK 1903.7.1.2203 from Burma; **B**. Syntype NHMUK 1906.4.4.18a from Akouktoung; **C**. Syntype NHMUK 1906.4.4.18 from Thondoung, near Mya; **D, E**. Syntype NHMUK 1888.12.4.217–219 from Akouktoung; **F, G**. *Diplommatina oligopleuris* Blanford, 1868: **F**. Syntype NHMUK 1903.7.1.2273 from Khasi; Teria Ghat; **G**. Specimen NHMUK 1903.7.1.2197 from Dafla Hills; Township: Dikrang Valley.

Myanmar • ***Syntypes***. NHMUK 1888.12.4.217–219 (3 shells; Fig. 18D, E) from Akouktoung, Pegu; Theobald coll. • ***Syntypes***. NHMUK 1906.4.4.18a (6 shells; Fig. 18B) from Akouktoung; W.T. Blanford coll. • ***Syntypes***. NHMUK 1906.4.4.18 (6 shells; Fig. 18C) from Thondoung, near Mya; W.T. Blanford coll.

##### Other material.

Myanmar • NHMUK 1903.7.1.2203 (1 shell; Fig. 18A) from Burma (figured in Godwin-Austen 1884: pl. 49, figs 6, 6a); Godwin-Austen coll.

##### Diagnosis.

Shell dextral, pupate-fusiform, translucent, and pale yellowish to whitish. Whorls **~**6, rounded; spire elongate-conical; suture wide, deep. Teleoconch with weak, irregularly spaced radial ribs. Last three whorls nearly equal in width; penultimate whorl slightly wider than others. Aperture ovate; columellaris strong, distinct, directed downward. Peristome double, expanded, multi-layered; inner peristome broadly expanded over outer peristome; lip little thickened, multi-layered. Umbilicus narrowly open.

##### Distribution.

The species is currently known from its type locality near the border of the present-day Bago and Ayeyarwady regions. The Akauk Taung Hill (18°30'33.7"N, 95°06'48.5"E) is located in Pyay District, Bago Region, while Thondoung and Yenangdoung likely correspond to the area of Yae Nan Taung Village, south of Akauk Taung Hill, in Hinthada District, Ayeyarwady Region.

In addition, the species has been reported from several other localities in Myanmar: Henzada (Hinthada City, Ayeyarwady Region), Thayet Myo (Thayet City, Thayet District, Magway Region), Pegu (Bago Region), Tongoop, Arakan (Taungup Township, Thandwe District, Rakhine State), and near Moulmein (Theobald and Stoliczka 1872; Nevill 1878; Gude 1921). It has also been reported from Meghalaya State, India (Ramakrishna et al. 2010).

##### Remarks.

In the original description, Blanford (1865: 85) did not indicate the number of type specimen lots. However, the stated collection locality suggests that the type series comprised at least three lots from distinct collecting sites: ‘Akoutoung’ (= Akauk Taung), Pyay District, Bago Region; ‘Thondoung’, a hill south of Akauk Taung, Hinthada District, Ayeyarwady Region; and ‘Yenandoung’ (= Yae Nan Taung), Hinthada District, Ayeyarwady Region. The NHM collection holds three lots of this species, all originating from W.T. Blanford’s collections: NHMUK 1888.12.4.217–219 from Akauk Taung, Pegu; NHMUK 1906.4.4.18a from Akauk Taung; and NHMUK 1906.4.4.18 from Thondoung, near Mya. These three lots constitute the type series and are herein recognised as syntypes. Representative specimens from each syntype lot are also illustrated in this study.

#### 24 Diplommatina oligopleuris

Taxon classificationAnimaliaArchitaenioglossaDiplommatinidae

Blanford, 1868

29C0E79C-5290-5241-A607-ECD8408BA111

Fig. 18F, G

Diplommatina oligopleuris Blanford, 1868: 82, pl. 3, fig. 4. Type locality: Teria Ghat ad latus meridionale montium Khasi. Hanley and Theobald 1875: 49, pl. 119, figs 2, 3. Pfeiffer 1875: 74. Theobald 1876: 42. Nevill 1878: 285. Godwin-Austen 1884: pl. 50, fig. 1. Gude 1921: 325, 326.
Diplommatina (Eudiplommatinu) oligopleuris —Kobelt and Möllendorff 1898: 137.
Diplommatina *olygopleuris* [sic]—Theobald and Stoliczka 1872: 330, 331.
Diplommatina (Diplommatina) oligopleuris —Kobelt 1902: 440.

##### Type material examined.

India • ***Syntype***. NHMUK 1903.7.1.2273 (1 shell; Fig. 18F) from Teria Ghat, Khasi; Godwin-Austen coll.

##### Other material.

• NHMUK 1903.7.1.2197 (1 shell; Fig. 18G) from Dikrang Valley, Dafla Hills; Godwin-Austen coll. NHMUK 1903.7.1.2274 (7 shells) from Moyong, N.W. Khasi; Godwin-Austen coll. NHMUK 1903.7.1.2275 (9 shells) from Rywuk, Garo Hills; Godwin-Austen coll. NHMUK 1903.7.1.273 (37 shells) from Khasi Hills; Godwin-Austen coll.

##### Diagnosis.

Shell dextral, ovate-fusiform, translucent, pale yellowish to whitish. Whorls ~5–6, well rounded; spire depressed-conical; suture wide, deep. Teleoconch with weak, widely and equally spaced radial ribs throughout. Penultimate whorl well rounded, much wider than preceding and last whorls. Aperture circular; columellaris weak or strong, directed downward. Peristome expanded, double, multi-layered; outer peristome slightly broader than inner peristome. Umbilicus closed.

##### Distribution.

This species was originally discovered from Teria Ghat, Khasi Hills and later reported from Kumah Hill and Baom in Rakhine State, Myanmar (Theobald and Stoliczka 1872: 330).

#### 25 Diplommatina polypleuris

Taxon classificationAnimaliaArchitaenioglossaDiplommatinidae

Benson, 1857

64984CA9-EF1D-50D7-AE57-A9D5249DDC56

Fig. 19A, B

Diplommatina polypleuris Benson, 1857: 203. Type locality: ad Nancla. Theobald 1858: 318; Pfeiffer, 1858: 11. Godwin-Austen 1868: 83, pl. 3, fig. 1. Theobald and Stoliczka 1872: 330. Hanley and Theobald 1875: 56, pl. 140, fig. 10. Pfeiffer 1875: 73. Theobald 1876: 42. Nevill 1878: 285. Godwin-Austen 1884: pl. 45, figs 1, 1a. Godwin-Austen 1886: 176. Gude 1921: 328.
Diplommatina polypleuris var. Godwin-Austen, 1870: 4, pl. 1, figs 4, 4a. Type locality: North Cachar and north Jaintia hills [mountainous region in Assam and Meghalaya state, India].
Diplommatina polypleuris var. minuta Godwin-Austen, 1876: 178 (nomen nudum).
Diplommatina polypleuris var. minuta Godwin-Austen, 1886: 176 [in the name usage list].
Diplommatina (Eudiplommatina) polypleuris —Kobelt and Möllendorff 1898: 137.
Diplommatina (Diplommatina) polypleuris —Kobelt 1902: 441. Preece et al. 2022: 86, fig. 34e.

##### Type material examined.

• ***Syntype***. UMZC I.102640 (1 shell), no locality data; R. McAndrew coll.

**Figure 19.**
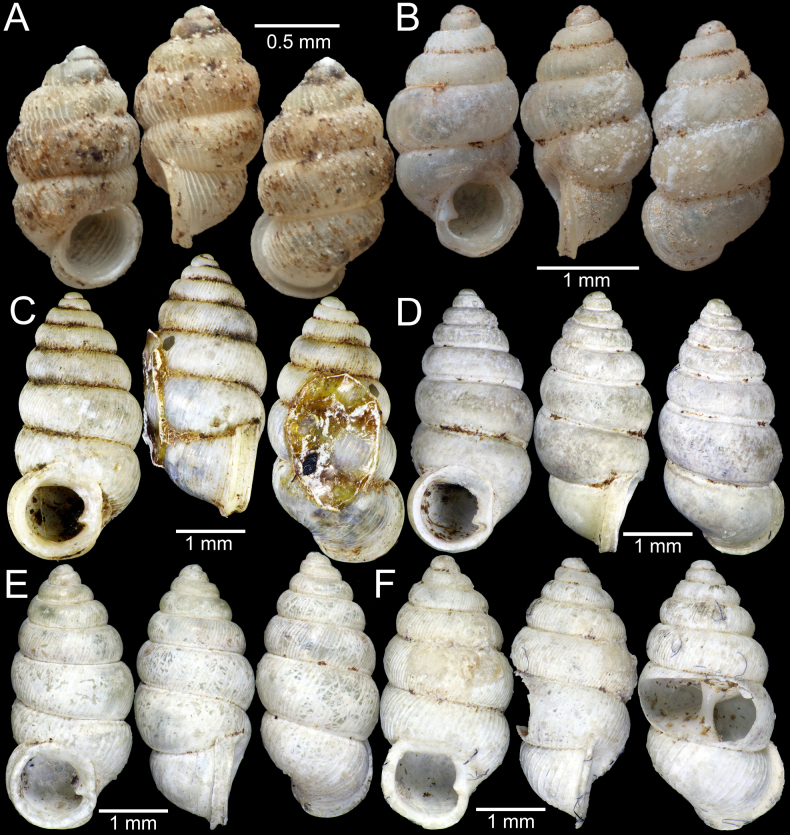
**A, B**. *Diplommatina polypleuris* Benson, 1857: **A**. Specimen NHMUK 1903.7.1.2252 from Nongtung, Khasi; **B**. Specimen NHMUK 1891.3.17.728–730 from Nattoung; **C–F**. *Diplommatina pupaeformis* Theobald, 1870: **C**. Specimen NMW 1955.158.27985 from Upper Salwin, Shan State; **D–F**. Possible syntype NMW 1894.015.00102 from Shan State.

##### Other material.

• NHMUK 1903.7.1.2314 (2 shells) from Jawai, Jaintia; Godwin-Austen coll. NHMUK 1903.7.1.2177 (1 shell) from Khasi (figured in Godwin-Austen 1884: pl. 45, figs 1, 1a); Godwin-Austen coll. NHMUK 1903.7.1.2269 (24 shells) from Shillong, Khasi Hills; Godwin-Austen coll. NHMUK 1905.5.5.49 (12 shells) from North Scarp, Khasi Hills; W.T. Blanford coll. UMZC I.102645 (1 shell), no locality data; R. McAndrew coll. NHMUK 1891.3.17.728–730 (3 shells; Fig. 19B) labelled as *D. polypleuris* var. from Nattoung; Sowerby coll. NHMUK 1903.7.1.2252 (5 shells; Fig. 19A) labelled as D. polypleuris var. minuta from Nongtung, Khasi; Godwin-Austen coll. NHMUK 1903.7.1.2262 (1 shell) labelled as D. polypleuris var. minuta from Japvo Peak, Naga; Godwin-Austen coll. NHMUK 1903.7.1.263 (1 shell) labelled as D. polypleuris var. minuta from Munken Valley, Jaintia; Godwin-Austen coll.

##### Diagnosis.

Shell dextral, ovate-fusiform, translucent, whitish to yellowish. Whorls ~5, rounded; spire depressed conical; suture wide, deep. Teleoconch with strong, densely and equally spaced radial ribs throughout. Last three whorls nearly equal in width; penultimate whorl slightly wider than others. Aperture rounded; columellaris weak, indistinct. Peristome expanded, double, multi-layered; outer peristome expanded beyond inner peristome. Umbilicus narrowly open.

##### Distribution.

This species was originally known from its type locality in Nanclai Poonjee, Assam, India, and was later recorded from the Dafla Hills, North Khasi Hills, and Darjeeling (Hanley and Theobald 1870; Gude 1921; Preece et al. 2022). In Myanmar, it has been reported from Nattoung, Sandoway, which likely corresponds to the Thandwe District area in Rakhine State (Theobald and Stoliczka 1872; Preece et al. 2022).

##### Remarks.

Godwin-Austen (1870: 4) recognised specimens from ‘North Cachar and North Jaintia Hills’ as a distinct variety, but did not provide a name for it. Subsequently, he introduced the name var. *minuta* for specimens from ‘Shengorh and Torúpútú Peaks’ (Godwin-Austen 1876: 178). However, this nomination lacks a definition, indication, description, and any reference to earlier work; therefore, this varietal name was not made available (ICZN 1999: Art. 12). Later, in the list of name usages under *D. polypleuris*, Godwin-Austen (1886: 176) stated ‘from N. Jaintia is distinct; I have named it *minuta*’, with an explicit indication referring to the published description in Godwin-Austen (1870). It seems that the name var. *minuta* was made available from Godwin-Austen (1886). Regarding the type series, the NHM holds one lot, NHMUK 1903.7.1.2252 (4 shells), registered as syntypes of *D. polypleuris minuta*. However, the recorded locality is from ‘Nongtung, Khasi’, which does not match the locality ‘North Jaintia Hills’ associated with the original description. Therefore, this specimen lot is unlikely to be the syntype, since it neither originates from the type locality nor appears to be part of the type series examined by Godwin-Austen.

The specimen figured in Preece et al. (2022: fig. 34e), labelled as *D. polypleuris*, appears to have prominent radial ribs and a distinct horizontal palatalis, whereas the specimens from Nattoung (Fig. 19B), referred to as *D. polypleuris* var., tend to have weaker radial ribs and a conspicuous vertical plica. These differences suggest that the two are unlikely to represent the same taxon.

#### 26 Diplommatina pupaeformis

Taxon classificationAnimaliaArchitaenioglossaDiplommatinidae

Theobald, 1870

BBD32EDF-393E-5876-ACC2-F2EE970D5C3D

Fig. 19 C–F

Diplommatina pupaeformis Theobald, 1870: 398. Type locality: Shan States. Pfeiffer 1875: 84, 85. Theobald 1876: 42. Godwin-Austen 1884: pl. 46, figs 4, 4a. Godwin-Austen 1886: 182. Gude 1921: 329.
Diplommatina salwiniana var. pupaeformis —Nevill 1878: 285.
Diplommatina (Eudiplommatina) pupaeformis —Kobelt and Möllendorff 1898: 137.
Diplommatina (Diplommatina) pupaeformis —Kobelt 1902: 442.

##### Type material examined.

Myanmar • **Possible *syntypes***. NMW 1894.015.00102 (3 shells; Fig. 19D–F) from Shan State; Fedden coll.

##### Other material.

Myanmar • NMW 1955.158.27985 (1 shell; Fig. 19C) from Upper Salwin, Shan State Godwin-Austen ex. Melvill-Tomlin coll.

##### Diagnosis.

Shell sinistral, fusiform to almost pupilliform, whitish or transparent yellow. Whorls ~7½ and slightly convex; spire conical; suture wide, deep. Protoconch ~1¾ whorl, nearly smooth, without radial ribs or with weak wrinkles and coalescing round malleated pits. Teleoconch with delicate costae then becoming strong, regularly spaced radial ribs towards aperture. No spiral striation. Penultimate whorl slightly wider than last whorl. Aperture rounded; columellaris distinct, slightly directed downward to palatal wall. Peristome broadly expanded; lip thickened, multi-layered. Umbilicus sealed.

##### Distribution.

This species is currently known only from Shan State and is likely endemic to the region.

##### Remarks.

This is the only one of the four diplommatinid species described by Theobald (1870) for which the type series could be located (T. White, B. Rowson, and J. Gallichan, pers. comm.). In the introduction, Theobald (1870: 398) stated that the specimens were obtained from Mr. Fedden, who had collected them after returning from the Upper Salwin. The NMW, Cardiff, holds a lot from the Fedden collection containing three shells, and with the collection locality labelled ‘Shan States, Burmah’, which are considered syntypes.

#### 27 Diplommatina puppensis

Taxon classificationAnimaliaArchitaenioglossaDiplommatinidae

Blanford, 1863

00AFCD96-C0BE-55B9-A047-AA85D1EC7DC8

Fig. 20A, B

Diplommatina puppensis Blanford, 1863: 324, 325, Type locality: Puppa Hill in Upper Burma. Godwin-Austen 1868: 84, pl. 4, figs 2, 2a. Hanley and Theobald 1875: 55, pl. 139, figs 8, 9. Pfeiffer 1875: 75, 76. Theobald 1876: 42. Nevill 1878: 284. Godwin-Austen 1884: pl. 49, fig. 9. Godwin-Austen 1886: 180, 181. Gude 1921: 329, 330.
Diplommatina *pappensis* [sic.]—Blanford 1864: 443.
Diplommatina (Eudiplommatina) puppensis —Kobelt and Möllendorff 1898: 137.
Diplommatina (Diplommatina) puppensis —Kobelt 1902: 442.

##### Type material examined.

**Figure 20.**
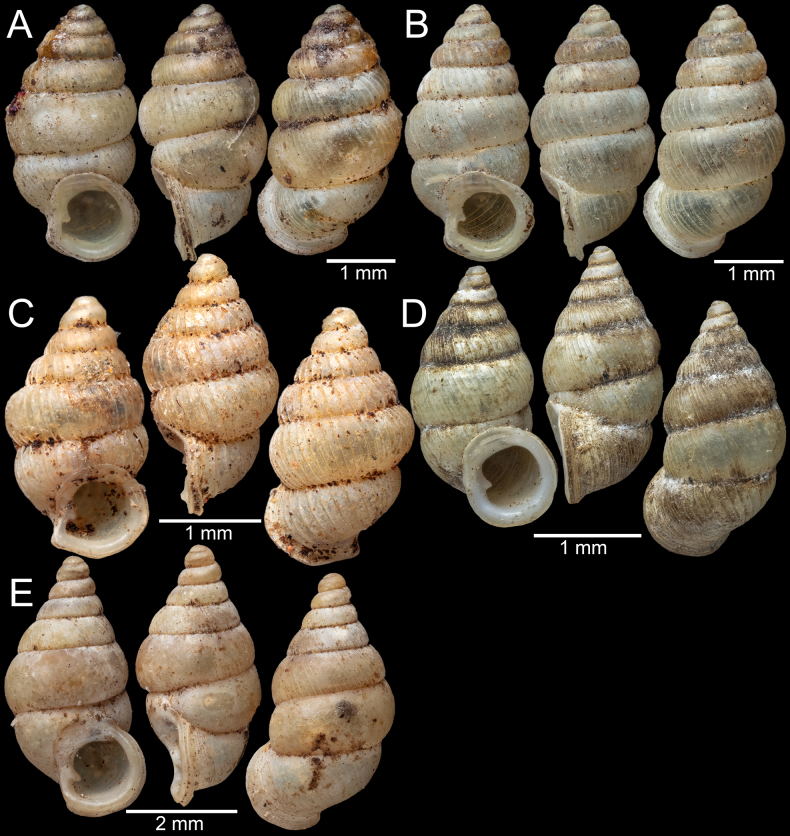
**A, B**. *Diplommatina puppensis* Blanford, 1863 from Puppa Hill, extinct volcano, Upper Burma: **A**. Syntypes NHMUK 1906.4.4.15; **B**. Syntype NHMUK 1903.7.1.2278; **C**. *Diplommatina sperata* Blanford, 1862, possible syntype NHMUK 1888.12.4.134–137 from Maii, Arakan Coast; **D**. *Diplommatina thomsoni* Godwin-Austen, 1892, syntype NHMUK 1903.7.1.2191 from South Burrail; **E**. Diplommatina tumida var. laisenensis Gude, 1921, syntype NHMUK 1903.7.1.2136 from Nongmaiching, Munipur.

Myanmar • ***Syntypes***. NHMUK 1906.4.4.15 (4 shells; Fig. 20A) from Puppa Hill, extinct volcano, Upper Burma; W.T. Blanford coll. • ***Syntype***. NHMUK 1903.7.1.2278 (1 shell; Fig. 20B) from Puppa Hill, extinct volcano, Upper Burma (figured in Godwin-Austen 1884: pl. 49, fig. 9); Godwin-Austen coll.

##### Diagnosis.

Shell dextral, conical-fusiform, translucent, whitish to pale yellowish. Whorls ~6–7, rounded; spire elongate-conical; suture wide, deep. Teleoconch slightly thin, with closely and equally spaced radial ribs throughout. Penultimate whorl slightly wider than last whorl. Aperture rounded; columellaris distinct, directed downward. Peristome expanded, double, multi-layered; basal edge slightly sinuous, protruded; inner peristome broadly expanded on parietal side. Umbilicus closed.

##### Distribution.

This species was originally known only from its type locality, ‘Puppa Hill, Ava’, which likely corresponds to present-day Mount Popa (20°55'N, 95°14'E) in central Mandalay Region.

#### 28 Diplommatina salwiniana

Taxon classificationAnimaliaArchitaenioglossaDiplommatinidae

Theobald, 1870

E9B28141-94A3-5A94-9E28-71E380F936F4

Diplommatina salwiniana Theobald, 1870: 398. Type locality: Shan States. Pfeiffer 1875: 84. Nevill 1878: 285. Gude 1921: 332.
Diplommatina *salewiniana* [sic.]—Theobald 1876: 42.
Diplommatina (Eudiplommatina) salwiniana —Kobelt and Möllendorff 1898: 138.
Diplommatina (Diplommatina) salwiniana —Kobelt 1902: 444.

##### Type material examined.

Unknown.

##### Diagnosis.

Shell sinistral, ovate-turreted, pale yellowish; suture impressed. Whorls 7½, convex, increasing regularly; radial ribs distinct, more widely spaced on last whorl. Aperture rounded-oval; columella straight; columellaris distinct.

##### Distribution.

The species is currently known only from its type locality in Shan State, Myanmar.

##### Remarks.

Among the four diplommatinid species described by Theobald (1870), the status of this species remains uncertain. The original description lacked an illustration, the type specimens could not be located (T. White, B. Rowson, and J. Gallichan, pers. comm.), and no subsequent illustrations have been published. The diagnosis provided here is based on the original description and the account by Gude (1921).

#### 29 Diplommatina sperata

Taxon classificationAnimaliaArchitaenioglossaDiplommatinidae

Blanford, 1862

DC7DF65F-026E-5A02-9F5D-3B1DF6C8D9F7

Fig. 20C

Diplommatina sperata Blanford, 1862: 143, 144. Type locality: in montibus Arakan a Pegu secernentibus. Pfeiffer 1865: 10. Godwin-Austen 1868: 84, pl. 4, figs 1, 1a. Theobald and Stoliczka 1872: 330. Theobald 1876: 42. Nevill 1878: 284. Godwin-Austen 1884: pl. 46, figs 5, 5a. Godwin-Austen 1886: 179. Gude 1921: 335, 336. Budha et al. 2017: 22, figs 8b, 9n.
Diplommatina (Eudiplommatina) sperata —Kobelt and Möllendorff 1898: 138.
Diplommatina (Diplommatina) sperata —Kobelt 1902: 446. Budha et al. 2015: 7.

##### Type material examined.

Myanmar • **Possible *syntypes***. NHMUK 1888.12.4.134–137 (4 shells; Fig. 20C) from Maii, Arakan Coast; W. Theobald coll.

##### Diagnosis.

Shell dextral, ovate-fusiform, translucent, pale yellowish or whitish. Whorls ~5–6, well rounded; spire depressed-conical; suture wide, deep. Teleoconch with wide, equally spaced radial ribs throughout. Penultimate whorl well rounded, wider than last whorl. Aperture ovate; columellaris prominent, directed downward. Peristome expanded, double, multi-layered; basal edge slightly sinuous and protruded; inner peristome broadly expanded on parietal side. Umbilicus closed.

##### Distribution.

This species was originally known from its type locality in the Arakan Hills, Bago Region, Myanmar. It was later recorded from ‘Moditoung’, likely corresponding to the Modi Taung Hills in Padauang Township, Pyay District, Bago Region, and from Mai-i, likely in the area of Ma-ei Town, Thandwe District, Rakhine State (Theobald and Stoliczka 1872). More recently, it has been reported from Nepal (Kuznetsov and Schileyko 1997; Budha et al. 2015, 2017).

##### Remarks.

This species has also been reported from several localities in southwestern to eastern Myanmar, including Ayeyarwady, Bago, and Tanintharyi regions (Blanford 1865).

It appears to have a broad distribution across Bangladesh, India, and Nepal (Budha et al. 2015; Preece et al. 2022). However, Budha et al. (2017) noted that specimens from Nepal tend to have thinner shells and much thinner peristomal lips than the syntypes, and are therefore provisionally assigned to this species.

#### 30 Diplommatina thomsoni

Taxon classificationAnimaliaArchitaenioglossaDiplommatinidae

Godwin-Austen, 1892

6A512F5A-D752-58F8-B68B-44D7CC890105

Fig. 20D

Diplommatina thomsoni Godwin-Austen, 1892: 514. Type locality: South Burrail. Godwin-Austen 1897: 9, 10, pl. 65, figs 2, 2a. Gude 1921: 344. McGhie 2008: 32.
Diplommatina (Metadiancta) thomsoni —Kobelt and Möllendorff 1898: 139. Kobelt 1902: 451.

##### Type material examined.

India • ***Syntypes***. NHMUK 1903.7.1.2191 (14 shells; Fig. 20D) from South Burrail; Godwin-Austen coll.

##### Diagnosis.

Shell dextral, ovate-fusiform, translucent, whitish. Whorls ~7, well rounded; spire elevated conical; suture wide, shallow. Teleoconch: earlier whorls with strong, narrowly and equally spaced radial ribs; penultimate and last whorls with slightly wider-spaced radial ribs. Penultimate whorl convex, slightly wider than last whorl. Aperture circular; columellaris weak, directed downward. Peristome expanded, double, multi-layered; inner peristome slightly broader than outer peristome; parietal callus thickened. Columella straight; umbilicus closed.

##### Distribution.

This species is known from its type locality in the Barail Range, Manipur State, India. Records from Myanmar, however, lack precise locality data (Gude 1921), and the presence there has not yet been reconfirmed.

##### Remarks.

There are three syntypes housed in The Manchester Museum, University of Manchester (EE.3511/130) (McGhie 2008).

#### 31 Diplommatina tumida laisenensis

Taxon classificationAnimaliaArchitaenioglossaDiplommatinidae

Gude, 1921

6B552319-D7BF-5B00-BFDA-8E82EA44EFC3

Fig. 20E

Diplommatina tumida var. Godwin-Austen, 1892: 512. Type locality: Laisen Peak and Trigonometrical Station, Munipur. Godwin-Austen 1897: 7, 8, pl. 64, fig. 7.
Diplommatina tumida var. laisenensis Gude, 1921: 338. Type locality: Burma: Laisen Peak and Nongmaiching Trigonometrical Station, Munipur.

##### Type material examined.

India • ***Syntypes***. NHMUK 1903.7.1.2139 (5 shells) labelled as *D. tumida* var. from Laisen H.S. Munipur; Godwin-Austen coll. • ***Syntypes***. NHMUK 1903.7.1.2136 (2 shells; Fig. 20E) labelled as *D. tumida* small var. from Nongmaiching, Munipur (figured in Godwin-Austen 1897: pl. 64, fig. 7); Godwin-Austen coll.

##### Diagnosis.

Shell dextral, ovate-fusiform, dull, whitish. Whorls ~7, well rounded; spire elevated conical; suture wide, shallow. Teleoconch: earlier whorls with weak, narrowly and equally spaced radial ribs; penultimate and last whorls with slightly wider-spaced radial ribs to nearly smooth. Penultimate whorl convex, wider than last whorl. Aperture circular; columellaris strong, directed downward. Peristome expanded, double, multi-layered; outer peristome slightly broader than inner peristome; parietal callus expanded. Columella straight; umbilicus closed.

##### Distribution.

This species is known from its type localities at Laisen Peak and the Trigonometrical Station in Manipur State, India. Records from Myanmar, however, lack precise locality data (Gude 1921), and the presence there has not yet been reconfirmed.

##### Remarks.

This subspecies was originally described in detail by Godwin-Austen (1892) under the designation ‘var.’ and later illustrated by Godwin-Austen (1897), although no formal name was proposed at that time. Gude (1921) subsequently republished the original description and introduced ‘*laisenensis*’ as a nomen novum, which has been made available. The type series of this subspecies corresponds to the specimens referred to as ‘var.’ and the ‘illustrated specimen’ by Godwin-Austen (1892, 1897) from Lisein Peak and the Nongmaiching Trigonometrical Station, Munipur. The NHM, London, holds two specimen lots considered syntypes: one containing five shells labelled ‘*Diplommatina tumida* var. from Laisen H.S., Munipur’, and another containing two shells labelled ‘*Diplommatina tumida* small var. from Nongmaiching, Manipur’.

## Conclusions

No taxonomic work on the microsnail genus *Diplommatina* in Myanmar has been conducted since the compilation by Gude (1921). Consequently, the species diversity of this genus has remained largely unchanged and unrevised for more than a century. Distributional information for many species is imprecise, as Gude (1921) listed nine species originally described from outside Myanmar as occurring in the country without providing precise locality data. The present study documents a total of 31 nominal species of *Diplommatina* from Myanmar, comprising 26 previously recorded species (Gude 1921), three newly recorded species (Panha and Burch 1998, 2005; Panha et al. 2002), and two newly described species. More accurate distribution ranges and collection localities are confirmed for *D. carneola* and *D. richthofeni* from Mon State, and *D. scalaroidea* from the Mandalay Region.

This survey also records three species newly reported from Myanmar that were originally described from Thailand: *D. akron* from Shan State, *P. suratensis* from Tanintharyi Region, and *D. nimanandhi* from Mon State. In addition, two new species discovered in this study represent the first *Diplommatina* species described from Myanmar in the twenty-first century: *D. prolixa* sp. nov. from Rathye Pyan Cave, Kayin State, and *D. somsakpanhai* sp. nov. from Buddha Cave, Tanintharyi Region. Both species appear to exhibit a high degree of endemism and are associated with limestone ecosystems along the Tenasserim Range.

For the remaining 23 previously recorded species, no new specimens were obtained in the present study, and here in we catalogued the type material of 21 of these species. This synoptic list is based on the type and authenticated specimens housed in the NHM, London, and is complemented with the type material of W. Theobald preserved in the NMW, Cardiff. Among these, nine species, namely *D. ambigua*, *D. animula*, *D. butleri*, *D. commutata*, *D. compacta*, *D. lapillus*, *D. munipurensis*, *D. thomsoni*, and *D. tumida laisenensis*, were provisionally listed as occurring in Myanmar by Gude (1921). However, these species were originally described by Godwin-Austen (1892) from either ‘Munipur’ or the ‘Naga Hills’. The locality name ‘Munipur’ refers to a region that was formerly part of the Burmese Empire and came under British control following the First Anglo-Burmese War (1824–1826), whereas the ‘Naga Hills’ are primarily located in northeastern India, although their ranges extend into the Naga Self-Administered Zone and Sagaing Region of Myanmar. Despite this geographical proximity, there is no definitive evidence confirming the occurrence of these taxa within the current political boundaries of Myanmar. Nevertheless, because type specimens are of critical importance for facilitating taxonomic revisions, validating species identities, and establishing a reliable taxonomic framework, these nominal species are included in the present study. Based on the currently available evidence, a total of 31 *Diplommatina* species are provisionally recognised from Myanmar. However, given the limited number of surveyed localities, the actual species richness is likely to be considerably higher. Additional, systematic field surveys are therefore necessary to improve our understanding of the diversity and biogeography of the land snail fauna in Myanmar and neighbouring regions.

Micro-computed tomography (micro-CT) is a highly effective technique for revealing important diagnostic characters, such as internal folds and lamellae, particularly in small-sized snails that are difficult to manipulate using conventional methods (e.g., Budha et al. 2017; Greke 2017). The utility of this tool has been demonstrated in several studies assessing internal shell characters across various diplommatinid genera (e.g., Liew et al. 2014; Liew and Schilthuizen 2016; Bochud et al. 2021).

## Supplementary Material

XML Treatment for Diplommatina

XML Treatment for Diplommatina scalaroidea

XML Treatment for Diplommatina carneola

XML Treatment for Diplommatina richthofeni

XML Treatment for Diplommatina akron

XML Treatment for Diplommatina nimanandhi

XML Treatment for Diplommatina prolixa

XML Treatment for Diplommatina somsakpanhai

XML Treatment for Pagodapalaina

XML Treatment for Pagodapalaina suratensis

XML Treatment for Diplommatina affinis

XML Treatment for Diplommatina ambigua

XML Treatment for Diplommatina angulata

XML Treatment for Diplommatina animula

XML Treatment for Diplommatina butleri

XML Treatment for Diplommatina commutata

XML Treatment for Diplommatina compacta

XML Treatment for Diplommatina crispata

XML Treatment for Diplommatina edentula

XML Treatment for Diplommatina exilis

XML Treatment for Diplommatina exserta

XML Treatment for Diplommatina henzadaensis

XML Treatment for Diplommatina lapillus

XML Treatment for Diplommatina munipurensis

XML Treatment for Diplommatina nana

XML Treatment for Diplommatina oligopleuris

XML Treatment for Diplommatina polypleuris

XML Treatment for Diplommatina pupaeformis

XML Treatment for Diplommatina puppensis

XML Treatment for Diplommatina salwiniana

XML Treatment for Diplommatina sperata

XML Treatment for Diplommatina thomsoni

XML Treatment for Diplommatina tumida laisenensis

## Additional information

### Artificial Intelligence (AI) use

The authors accept full responsibility for the content of the manuscript, including the disclosure of any use of AI.

No AI tools were used in the preparation of this manuscript.

### Author ORCIDs

P. Tongkerd https://orcid.org/0000-0001-9221-9293

N. Lwin https://orcid.org/0000-0002-4394-3741

J.D. Ablett https://orcid.org/0000-0002-7277-1934

A. Pholyotha https://orcid.org/0000-0001-6677-1164

S. Annate https://orcid.org/0009-0001-6359-7954

C. Sutcharit https://orcid.org/0000-0001-7670-9540

### Data availability

All of the data that support the findings of this study are available in the main text or Supplementary Information.

## Supplementary materials

Supplementary material 1 Supplementary information Data type zip Explanation note 360-degree rotatable 3D shell models of seven *Diplommatina* spp. and *Pagodapalaina suratensis*. https://binary.pensoft.net/file/1718380This dataset is made available under the Open Database License (http://opendatacommons.org/licenses/odbl/1.0/). The Open Database License (ODbL) is a license agreement intended to allow users to freely share, modify, and use this Dataset while maintaining this same freedom for others, provided that the original source and author(s) are credited. Piyoros Tongkerd, Ngwe Lwin, Jonathan D. Ablett, Arthit Pholyotha, Supunya Annate, Chirasak Sutcharit
